# Supplementary material for: Low-k nano-dielectrics facilitate electric-field induced phase transition in high-k ferroelectric polymers for sustainable electrocaloric refrigeration
Source: Nat Commun. 2024 Jan 24;15:702. doi: 10.1038/s41467-024-44926-8 (PMC10808131; doi:10.1038/s41467-024-44926-8)
Supplement: Supplementary file 1 — Supplementary Information [file 41467_2024_44926_MOESM1_ESM.pdf]

Supplementary Materials for

Low-k nano-dielectrics facilitate electric-field induced phase transition in high-k ferroelectric polymers for sustainable electrocaloric refrigeration

Qiang Li[1], Luqi Wei[2], Ni Zhong[2], Xiaoming Shi[3], Donglin Han[1], Shanyu Zheng[1], Feihong Du[1], Junye Shi[1], Jiangping Chen[1], Houbing Huang[3], Chungang Duan[2], Xiaoshi Qian[1,4*]

[1]State Key Laboratory of Mechanical System and Vibration, Interdisciplinary Research Center, Institute of Refrigeration and Cryogenics, and MOE Key Laboratory for Power Machinery and Engineering, School of Mechanical Engineering, Shanghai Jiao Tong University, Shanghai, 200240, China

[2]Key Laboratory of Polar Materials and Devices, Ministry of Education, Shanghai Center of Brain-inspired Intelligent Materials and Devices, East China Normal University, Shanghai, 200241, China

[3]School of Materials Science and Engineering and Advanced Research Institute of Multidisciplinary Science, Beijing Institute of Technology, Beijing, 100081, China

[4]Shanghai Jiao Tong University ZhongGuanCun Research Institute, Liyang, 213300, China

*Correspondence: xsqian@sjtu.edu.cn

Contents

1. Materials and Methods....3
  1.1. Sample preparation....3
  1.2. Electrocaloric refrigeration....3
  1.3. Reduction of thermal resistance....4
2. Characterization of EC polymers....5
  2.1. Measurement of specific heat....5
  2.2. Electrical breakdown field....5
  2.3. Mechanical properties....6
  2.4. The characterization method of electrocaloric effect (Direct measurement of entropy change)....7
  2.5. Infra-red camera for material temperature change....9
  2.6. Latent heat and phase change temperature measurement....12
  2.7. WAXD and SAXS measurement....13
  2.8. Temperature-dependent permittivity and polarization-electric field loops 15
  2.9. Phase-field Simulation of polarization enhancement....19
  2.10. Electrostatic Force Microscopy....20
  2.11. Morphology of dispersed diamond nanoparticles and nanocomposite....21
  2.12. The simulation of electric field distortion....23
  2.13. The ECE of BNNS-incorporated nanocomposites....24
  2.14. Landau-Devonshire phenomenological theory....25
  2.15. Performance at high particle content....26
3. Numerical evaluation of the rotary EC refrigeration devices....30
  3.1. System structure and working principles....30
  3.2. Initial and boundary conditions....32
  3.3. Numerical evaluation of the rotary EC System....34
4. EC device as a standard platform....36
  4.1. Electrocaloric cooling with electrostatic actuation....36
  4.2. Comparison of device performance....36

1. Materials and Methods

1.1. Sample preparation

The base P(VDF-TrFE-CFE) terpolymers (*e.g.,* P(VDF-TrFE-CFE) 65/35/7.5 mol%) were synthesized using a suspension polymerization process by Piezotech (France). Initially, nanodiamond (ND, 3A Company) was dispersed in N, N-dimethylformamide (DMF) for 8 h (using ultrasonic cell pulverizer) and ice bath environment was used to keep the mixture temperature constant. The ND particles was homogeneously mixed with a solution of P(VDF-TrFE-CFE) in DMF. The resulting mixture was cast onto a clean glass slide and dried at 60 °C for 8 hours. Following the evaporation of the solvent, the composite films were carefully peeled off and subjected to annealing at 115 °C for 12 hours to enhance crystallinity and eliminate the remaining residual solvent. The thickness of the nanocomposite film was about 10 μm. As a comparison, the base polymeric films were made using the same solution casting method.

Gold electrodes were sputtered on both sides of the films for the electronic characterization. The silver wires were glued to the electrodes on both sides of the EC film by silver glue. The voltage was applied to the EC film through such silver wires.

1.2. Electrocaloric refrigeration

A comparison of the working principles of vapor compression refrigeration technology and EC refrigeration technology is shown in **Supplementary Fig. 1**. There are similarities and clear differences between them[1-4].

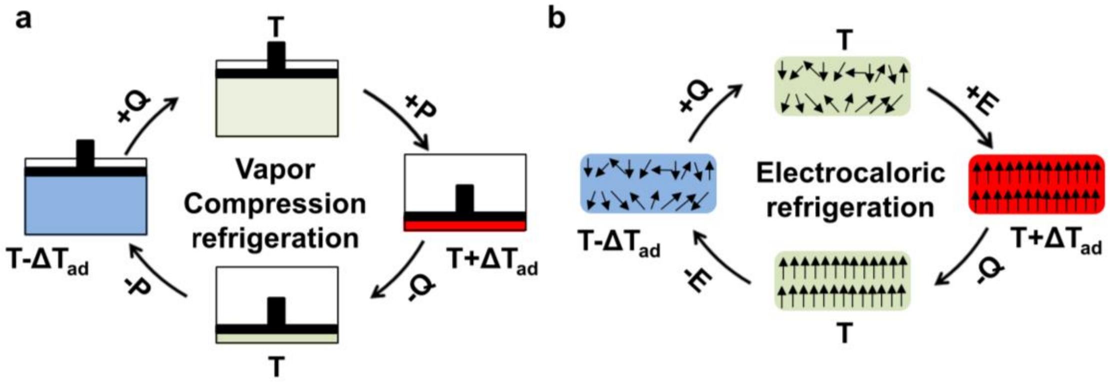

Supplementary Fig. 1. a-b Comparison between vapor compression refrigeration **(a)** and EC refrigeration **(b)**.

1.3. Reduction of thermal resistance

We prepared nanocomposites formed by incorporating ND with the base terpolymer. It's expected that the thermal conductivity of the ND-incorporated nanocomposites will be significantly higher than that of the base polymer. This is because we believe that the filling of ND helps to reduce the thermal resistance within the polymer, as shown in **Supplementary Fig. 2**.

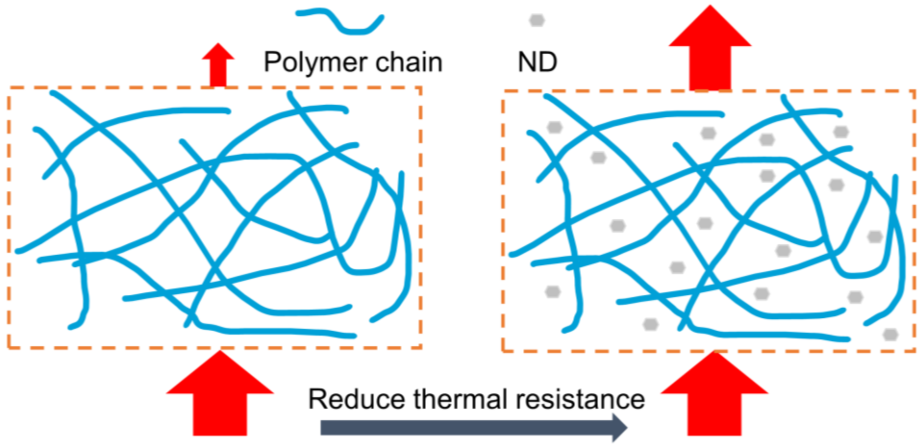

Supplementary Fig. 2. Diagram of the reduction of EC material thermal resistance by filling with ND.

2. Characterization of EC polymers

2.1. Measurement of specific heat

The specific heat of the base terpolymer and ND-incorporated nanocomposites were measured via the DSC 25 from TA Instruments. Three-step test was adopted to obtain the specific heat of such EC materials. As depicted in **Supplementary Fig. 3**, the specific heat of the EC materials remains almost unchanged over their operating temperature range (20-40 °C).

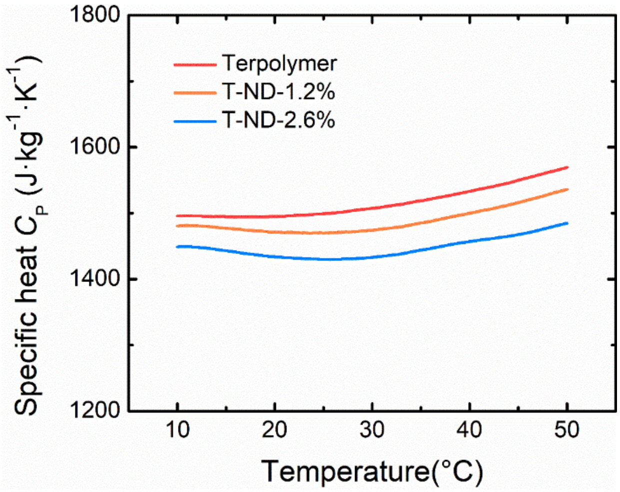

Supplementary Fig. 3. Specific heat of the base terpolymer and ND-incorporated nanocomposites at different temperatures.

The specific heat data of the base terpolymer and ND-incorporated nanocomposites was also used in the subsequent calculations of thermal conductivity, ECE and simulation of the designed EC devices.

2.2. Electrical breakdown field

The results of dielectric breakdown showing improved breakdown field of the ND-incorporated nanocomposites compared to that of the base polymer. Here, we developed a phase-field model to simulate the breakdown process of the base terpolymer and nanocomposites and the results are shown in **Supplementary Fig. 4**. T-ND-2.6% is less

susceptible to breakdown than the base terpolymer at the same electric field.

The breakdown field results from the phase-field simulation show that the introduction of low-k ND helps reduce the breakdown possibility of the polymeric matrix, *i.e.,* improve the breakdown field of nanocomposites when compared to the base terpolymer.

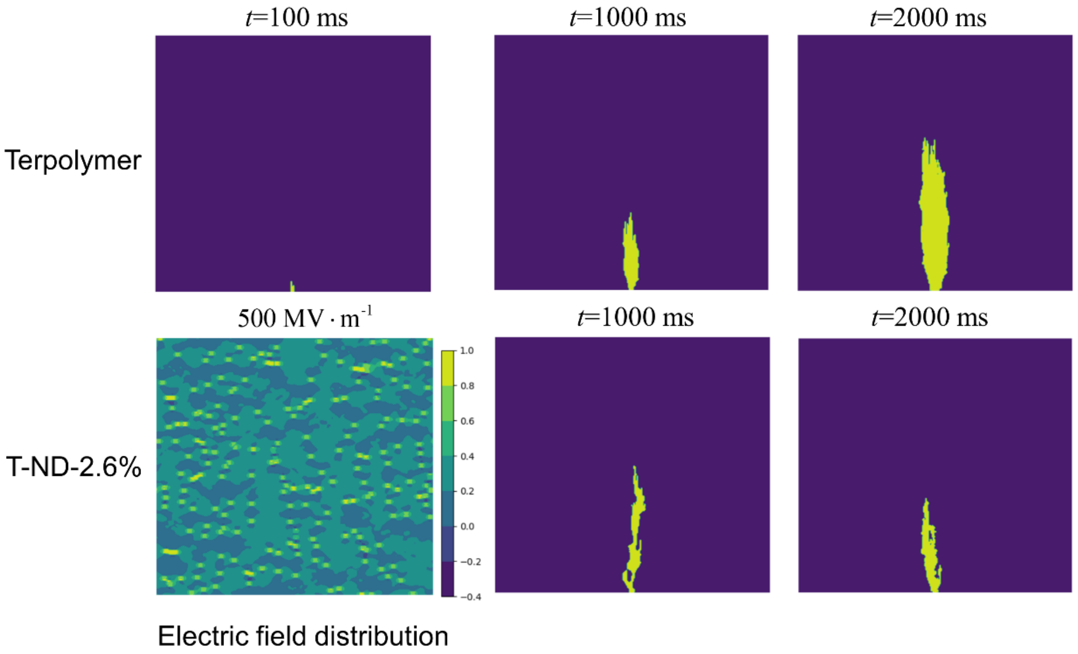

Supplementary Fig. 4. Breakdown morphologies of the base terpolymer and T-ND-2.6% from phase-field simulations.

2.3. Mechanical properties

Tensile mechanical testing was carried out via an Instron 5866 using a load cell (range of 200 N) and an extension rate of 10 mm·min^{-1}. The introduction of ND increased Young's modulus of the EC materials. As shown in **Supplementary Fig. 5**, the Young’s modulus of the EC materials increases with the increasing ND contents.

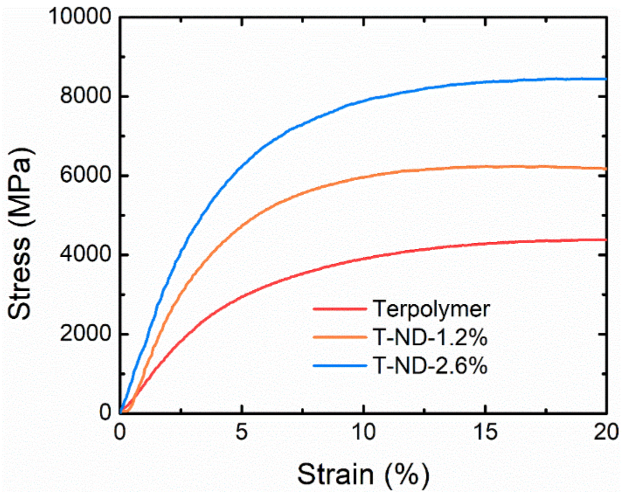

Supplementary Fig. 5. Stress-strain curves of the base terpolymer and nanocomposites.

2.4. The characterization method of electrocaloric effect (Direct measurement of entropy change)

The direct measurement method used in this experiment is a commonly-used method of ECE testing.[5] An EC calorimetry with *in-situ* calibration was proposed to adaptively measure the ECE albeit the variation of the case-sensitive thermal scenarios. The calibration is provided by a built-in reference heater (on the specific sample) that also serves as one of the electrodes. **Supplementary Fig. 6** is a schematic diagram of the homemade device for testing ECE in Shanghai Jiao Tong University.

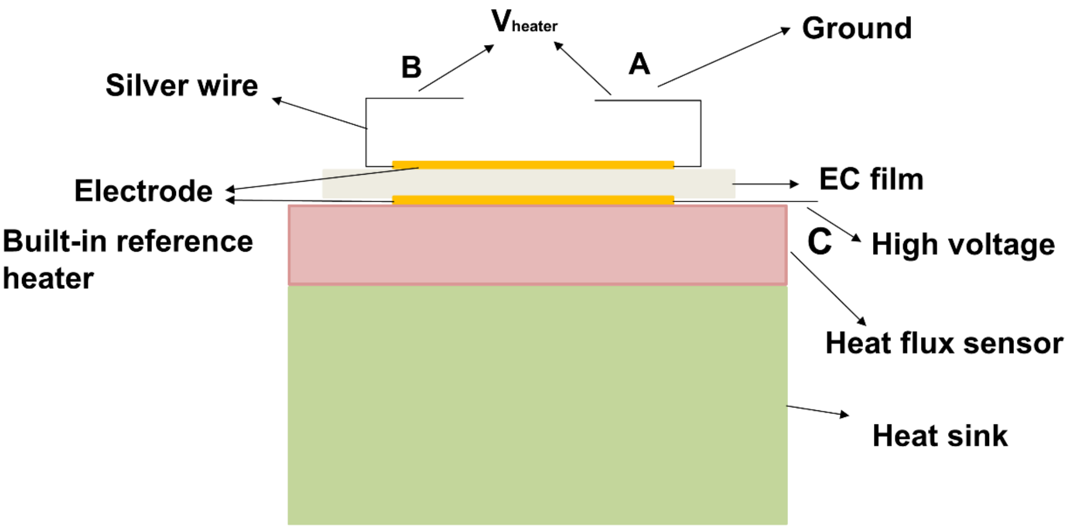

Supplementary Fig. 6. Schematic diagram of the device for testing ECE.

As depicted in **Supplementary Fig. 6**, two leads, denoted as A and C, are affixed to the top or bottom electrode of an Electrocaloric (EC) film. The EC film is securely positioned on the heat flux sensor. Known reference heat, generated through A and B leads with Resistor R, produces an electric signal via the heat flux sensor. Integrating the heat flux signal allows the determination of the ratio between the heat amount and the electric signal area for the sample. Subsequently, a high voltage (HV) is applied across the EC film (via A and C), leading to the ejection and absorption of a specific amount of heat, recorded by the sensor as electric signals. By integrating the EC signals and correlating them with the previously obtained ratio from the reference heat, calibrated EC-induced heat signals specific to this sample with its thermal contact to the sensor can be acquired. The detailed test process (including ECE and dielectric tests) can refer to these works[5-8].

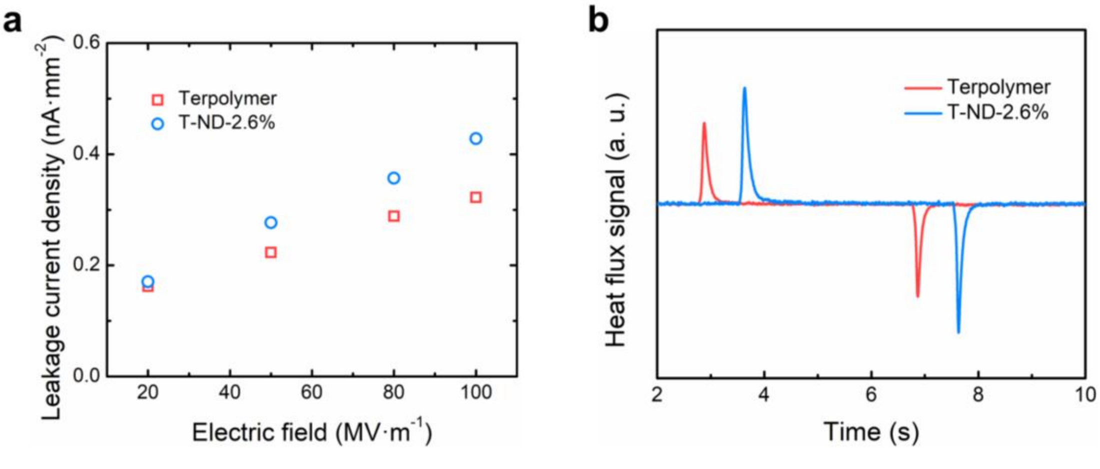

Supplementary Fig. 7. Leakage current density (a) and Heat flow intensity (b) of the base terpolymer and T-ND-2.6%.

As can be seen from the comparison of leakage current density (**Supplementary Fig. 7a**), T-ND-2.6% shows a very slight increase (< 30%) compared to the base terpolymer. No significant leakage currents appeared in either material. At 100 MV m^{-1}, the T-ND-

2.6% produces a Joule heating of 171.27 kJ m^{-3}, sharing less than 1% of the observed EC effect (28582.2 kJ·m^{-3}).

The results indicate that the T-ND-2.6% exhibited no obvious joule heating during the whole time of application of the electric field (100 MV·m^{-1}). Both the leakage current measurements and the heat flux signals of EC cycles confirm that the enhanced conduction loss by adding small amount of ND is limited and would not affect the EC performance of the materials.

In terms of material preparation, we are able to achieve excellent overall performance with an extremely small amount of ND doping. Here we compared the volumetric enhancement of ECE (the ratio between the EC enhancement in percentage and the volume percentage of the filler) at 100 MV·m^{-1} and RT, for different nano-fillers. As depicted in **Supplementary Fig. 8**, the ND introduced the highest volumetric EC enhancement (~23%/vol%) when compared with other fillers in the previous study[9-11].

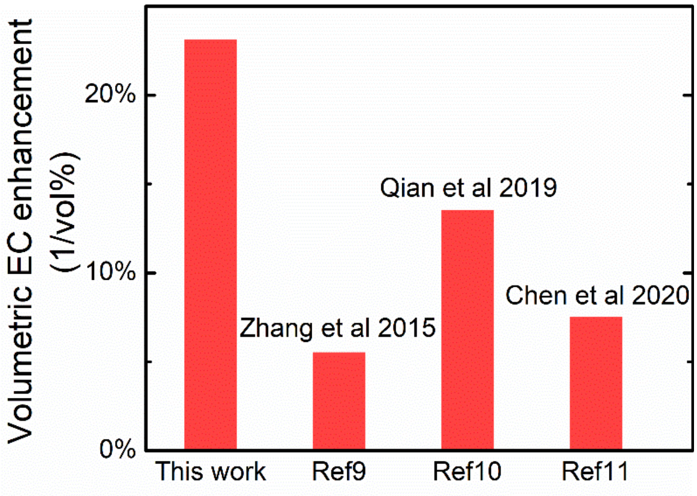

Supplementary Fig. 8. Volumetric EC enhancement of different fillers.

2.5. Infra-red camera for material temperature change

The above-mentioned homemade setup and the corresponding method has been

verified by many independent studies and groups[9, 12, 13]. To validate the specific EC measurement setup built in our facility at Shanghai Jiao Tong University, we further calibrated the ECE measurement instrument with an infra-red camera (**Supplementary Fig. 9**).

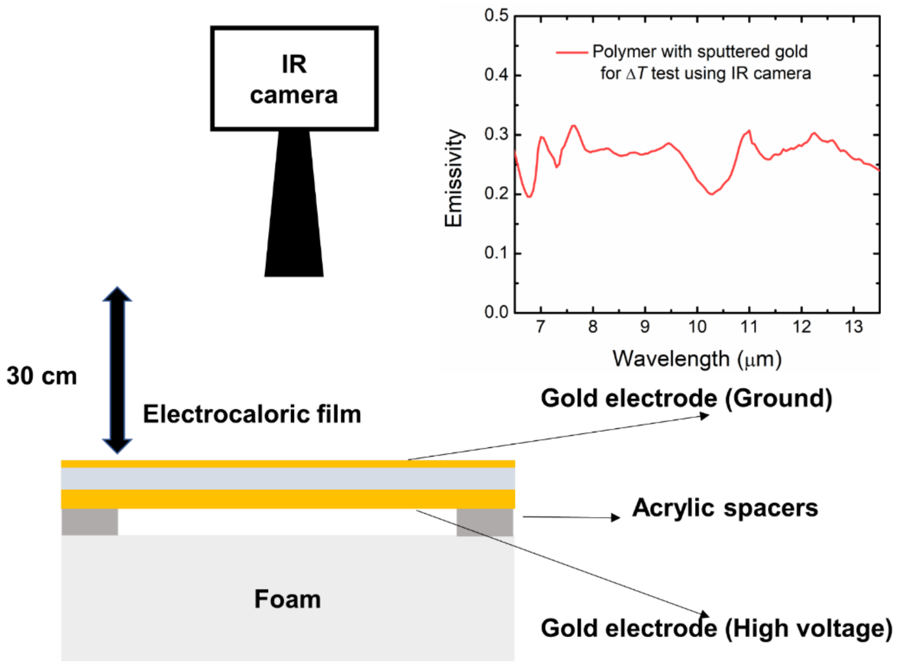

Supplementary Fig. 9. Schematic diagram of the IR camera for temperature changes of EC materials and the emissivity at mid-infrared wavelengths (7~13 μm).

Gold electrodes with a thickness of approximately 5 nm were sputtered on the top and bottom surfaces of the EC film. This ensures electrical conductivity while making it non-reflective under infrared cameras[14]. The emissivity of gold is much lower than the emissivity of the environment, and the test results show that the average emissivity of the EC film with sputtered gold at the mid-infrared wavelength (7-13 μm) is 0.27 (**Supplementary Fig. 9**). We used conductive silver epoxy to attach silver wires to the gold electrodes. Then we fixed the EC film onto the heat-insulating foam so that the lower surface could be regarded as heat-insulating. The IR camera (FLIR A655sc) was fixed 30 cm above the EC films[15].

In the direct measurement of Electrocaloric (EC) temperature change, a controlled ramping time of 0.1 s was employed for the application and removal of electric fields, as opposed to sudden changes. This deliberate choice deviates from strict adiabatic conditions, resulting in a recorded temperature change that is not a full representation of the 100% adiabatic temperature change (refer to **Supplementary Fig. 10** and **Supplementary Fig. 11**). The extended ramping duration serves the purpose of preventing the breakdown of EC films, facilitating temperature measurements at high electric fields.

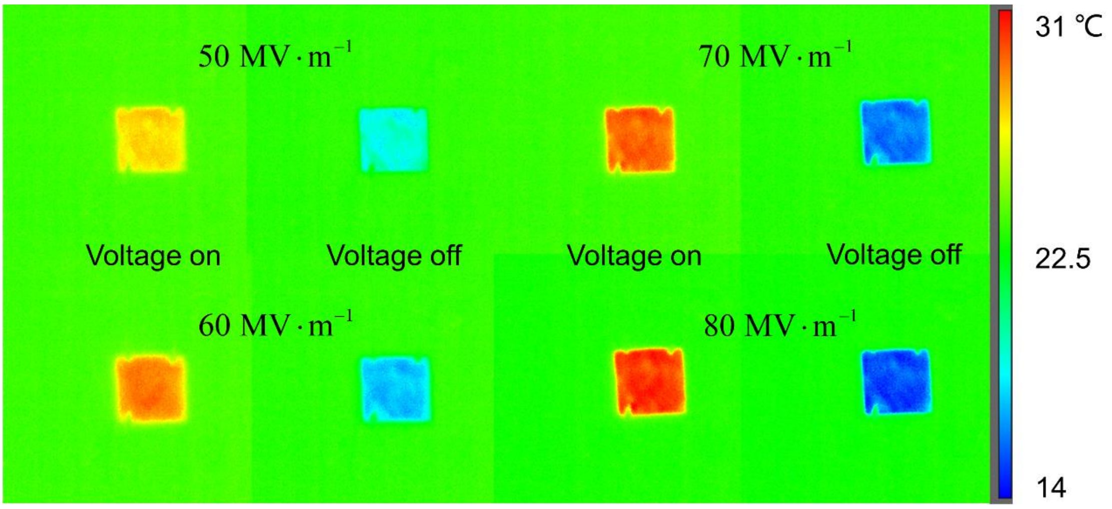

Supplementary Fig. 10. Direct temperature measurement of T-ND-2.6% via an IR camera, under different electric fields. The ambient temperature was kept at 22~23 ºC.

In fact, it is extremely hard to measure perfectly the isothermal entropy change (ΔS) or the adiabatic temperature change (ΔT). There is a difference between the temperature changes we measured utilizing the direct EC temperature-change measurement and IR camera but the difference isn't particularly large. Therefore, the ECE of T-ND-2.6% can be considered consistently better than that of the base terpolymer.

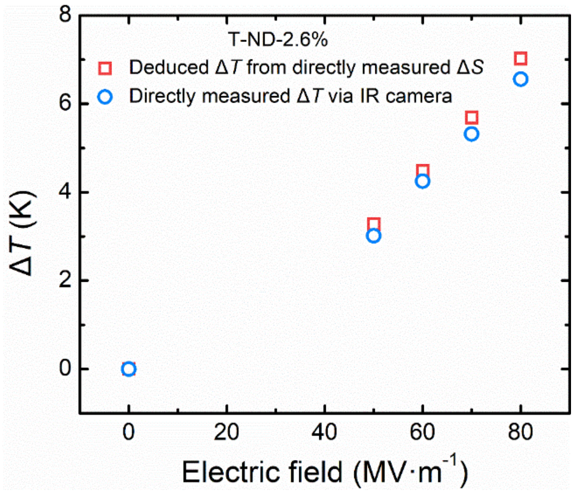

Supplementary Fig. 11. The directly measured ΔT and ΔS-deduced ΔT (T-ND-2.6%).

2.6. Latent heat and phase change temperature measurement

The determination of latent heat during the phase transitions of EC materials was conducted utilizing the DSC instrument (DSC25, TA Instruments). The experimental procedure involved an initial cooling step to -50 °C, followed by cyclic transitions between -50 °C and 160 °C at a ramp rate of 20 °C·min^{-1}. The DSC tests for the EC materials are shown in **Supplementary Fig. 12**. A comparison of the melting temperatures, and enthalpies can be obtained from the DSC results.

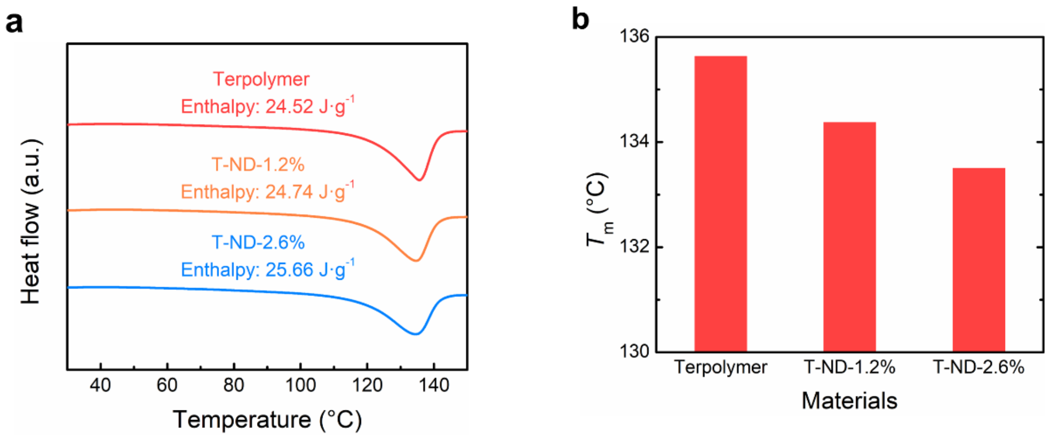

Supplementary Fig. 12. DSC profiles, melting temperature and enthalpy of the base terpolymer and nanocomposites. a DSC profiles of the samples recorded during the heating scan. b The melting temperatures (T_m) derived from the DSC profiles.

T_m of the nanocomposites decreases as the content of ND increases, indicating that the presence of nanoparticles could decrease the crystal size of the terpolymer[9]. This has been further confirmed by the WAXD data shown in **Fig. 2a-b**.

2.7. WAXD and SAXS measurement

In-situ Wide-angle X-ray diffraction (WAXD) and small-angle X-ray scattering (SAXS) characterizations were employed to monitor the structural evolution under different electric fields. WAXD and SAXS tests were conducted at the BL19U2 beamline of Shanghai Synchrotron Radiation Facility (SSRF), utilizing X-rays with a wavelength of 1.03 Å. The data acquisition time for each frame in both WAXD and SAXS was controlled at 15 seconds. The sample-to-detector distances were 207 mm for WAXD test and 2030 mm for SAXS test.

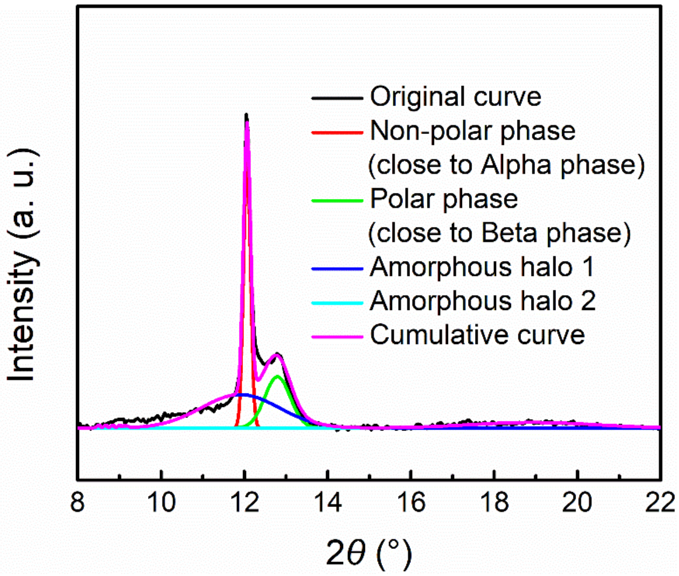

Supplementary Fig. 13. Estimates of crystallinity and fractions of non-polar, and polar phases.

The comparison of the SAXS and *in-situ* WAXD data for neat terpolymer and T-ND-2.6% before and after the application of electric field is shown in **Fig. 2a-e**. From these

data we can deduce the crystallinity and crystal sizes of such EC materials and the fraction of polar phase induced (**Supplementary Fig. 13** and **Supplementary Table 1**).

Compared to the base terpolymer, T-ND-2.6% have improved crystallinity and significantly reduced crystal size. The kind of composite can be induced to produce more polar-phase at the same electric field, as is shown in **Fig. 2f**. This could also explain the superior ECE of T-ND-2.6%.

Supplementary Table 1. Crystallinity, fraction of non-polar phases and polar phases, and crystal sizes of terpolymer and T-ND-2.6%

Sample	Terpolymer-0 MVm^{-1}	Terpolymer-50 MVm^{-1}	Terpolymer-80 MVm^{-1}	T-ND-2.6%-0 MVm^{-1}	T-ND-2.6%-50 MVm^{-1}	T-ND-2.6%-80 MVm^{-1}
Crystallinity	33%	41%	45%	36%	43%	48%
Field-induced polar phases	0%	8%	18%	0%	16%	31%
Crystal size of NP phases	35.4 nm	34.1 nm	32.4 nm	29.0 nm	26.5 nm	21.1 nm
Crystal size of polar-phases		8.8 nm	9.4 nm		12.1 nm	13.2 nm

The crystal types of the base terpolymer and PVDF are similar, but not identical. For example, the non-polar phase in P(VDF-TrFE-CFE) is not a complete TGTG structure, but can be considered non-polar. Therefore, it can be considered that the non-polar

phase is close to the α-phase and the polar phase is close to the β-phase.

We use the Lorentz-corrected SAXS spectra in the calculation of the long period. Evidence for crystal thinning in the chain direction is obtained from the small-angle X-ray scattering (SAXS) data presented in **Supplementary Fig. 14**, showing a correlation peak characterizing the period organization of the crystalline lamellae. The long period, L_P, which provides information on the periodic arrangement of the crystalline lamellae and amorphous regions. All the details of the SAXS analysis used in this study, for semi-crystalline polymers was already published[16].

$$L_p = \frac{2\pi}{q} \qquad \text{(Eq. S1)}$$

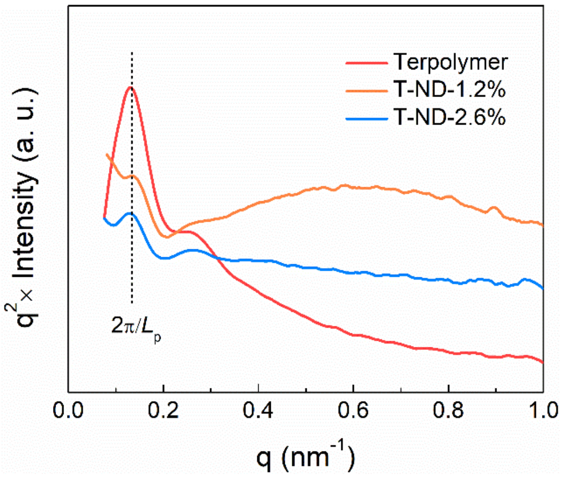

Supplementary Fig. 14. Lorentz-corrected SAXS spectra, $q^2 \cdot I(q)$, measured for the base terpolymer and ND-incorporated nanocomposites.

The reduction of the peak signal corresponding to the long cycle indicates that NDs may suppress the long-range ordering in the polymeric matrix.

2.8. Temperature-dependent permittivity and polarization-electric field loops

The permittivity spectroscopy were characterized using a precision LCR meter (HP 4284A) equipped with a temperature chamber. As shown in **Supplementary Fig. 15a-d**, the comparison shows that the dielectric properties of the ND-incorporated

nanocomposites differ very little from those of the base terpolymer owing to the low-k nature and the low content of ND.

The temperature-dependent permittivity revealed an outstanding feature as the content of ND increases. That is, in addition to a broad dielectric peak that progressively moves to high temperature with increasing frequency (a feature of a ferroelectric relaxor, indicated by the blue arrows in **Supplementary Fig. 15a-c**), a weak and relatively sharp dielectric peak appeared around 35℃, and the peak position seems frequency-independent.

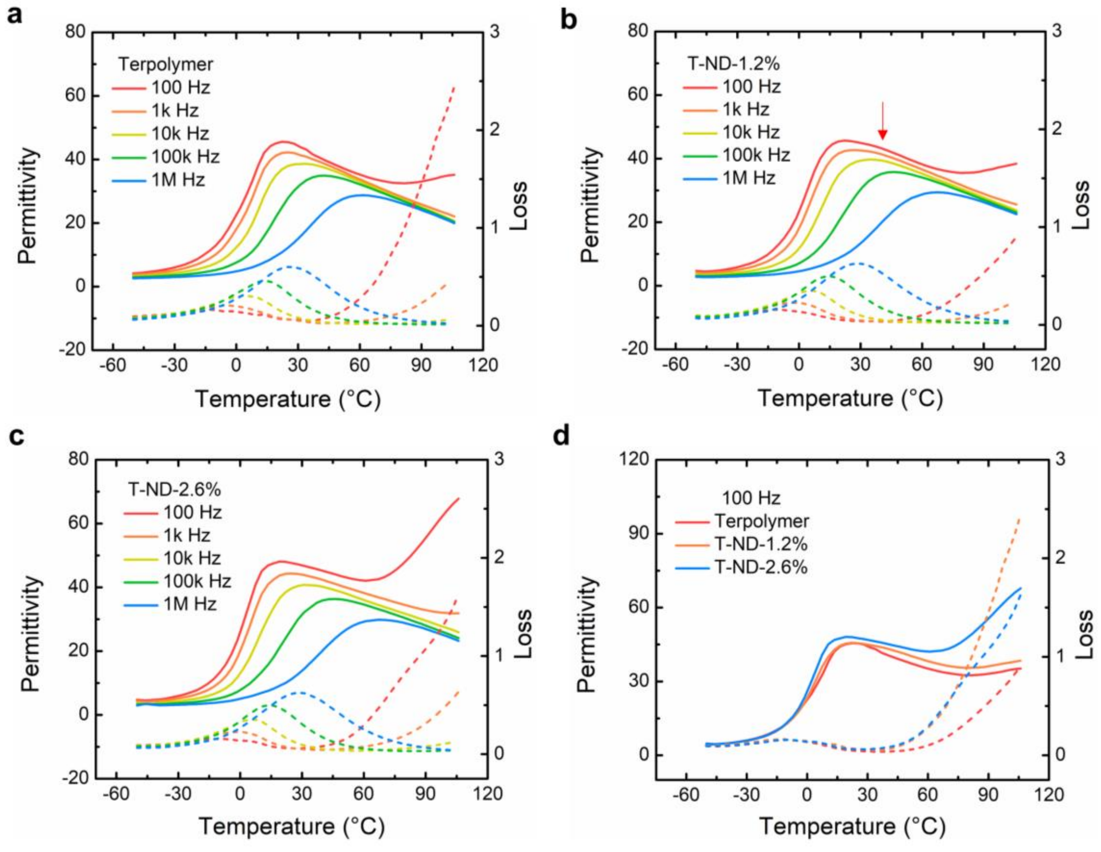

Supplementary Fig. 15. a-d Temperature-dependent permittivity and losses of the base terpolymer and nanocomposites. **a** Terpolymer. **b** T-ND-1.2%. **c** T-ND-2.6%. **d** Comparison of permittivity and losses of the base terpolymer and ND-incorporated nanocomposites at 100 Hz.

The polarization-electric (P-E) field loops of the terpolymer and nanocomposites were measured using a polarization loop and dielectric breakdown test system

developed by PolyK Technologies. Polarization-electric field loops (P-E loops) were measured using a modified Sawyer-Tower circuit at 10 Hz and room temperature (RT). Due to the existence of conduction loss, the tested P-E loops need to be further corrected[17].

The polymeric film is not an ideal insulator and leakage current is inevitable which induces conduction loss. In unipolar P-E loops, when the applied electric field is cycled once and returned to zero, the measured nominal polarization returns a finite nonzero value ΔP. For the PVDF-based terpolymer ferroelectric relaxor studied here, the contribution of polarization relaxation to the "remnant polarization" after an electric field cycle should be negligible[17], indicating that the observed nonzero ΔP is dominated by the conduction loss.

Furthermore, in our unipolar P-E loop measurement, through a test of decreasing the applied field frequency, we find a monotonous increase of ΔP(E=0), as shown in **Supplementary Fig. 16**. Such increase of ΔP(E=0) is consistent with the accumulation of charge leakage upon increasing the time duration, further confirming the nature of the conduction loss.

Therefore, by assuming that the ΔP is fully caused by the conduction and approximating a field-independent conductivity σ, the unipolar P-E loop follows:

$$\Delta P = \frac{2\int_0^{E_{\max}} \sigma E dE}{E_t} \quad \text{(Eq. S2)}$$

where E_t is the ramp rate of the applied electric field (the applied electric field is the unipolar triangular waveform), E is the applied electric field and E_{max} is the maximum applied electric field.

$$E = E_t * t \quad \text{(For the charging cycle) (Eq. S3)}$$

$$E = E_{\max} - E_t (t - T / 2) \quad \text{(For the discharging cycle) (Eq. S4)}$$

$$E_{\max} = E_t * T / 2 \quad \text{(Eq. S5)}$$

where t is the time at which the electric field E is applied and T is the period of the unipolar triangular applied electric field.

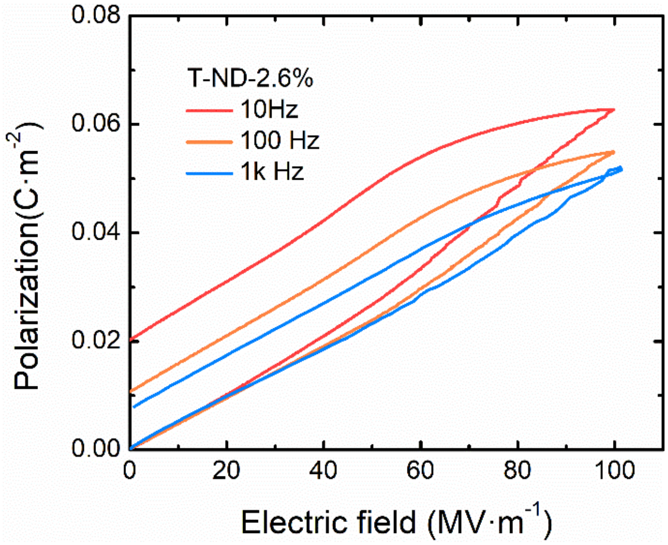

Supplementary Fig. 16. Unipolar P-E loops of T-ND-2.6% under different frequencies.

From Eq. S2, the conductivity σ can be calculated. Then the contribution of conduction to the polarization P_c under a certain electric field E_a on the P-E loops can be calculated as:

$$P_c = \int_0^{E_a} \sigma E dE / E_t \quad \text{(For the charging cycle) (Eq. S6)}$$

$$P_c = \int_0^{E_{\max}} \sigma E dE / E_t + \int_{E_a}^{E_{\max}} \sigma E dE / E_t \quad \text{(For the discharging cycle) (Eq. S7)}$$

Based on the above equations, the contribution from conduction to the P-E loops can be subtracted from the curves and the polarization returns to zero after the voltage is removed, as shown in **Fig. 3c**. The PE-loop results obtained from the test are shown in **Supplementary Fig. 17**.

After corresponding correction, the contribution from conduction to the P-E loops can be subtracted from the curves and the polarization returns to zero after the voltage is removed. After the conduction subtraction, the energy loss still exists, as shown in **Supplementary Fig. 17d**.

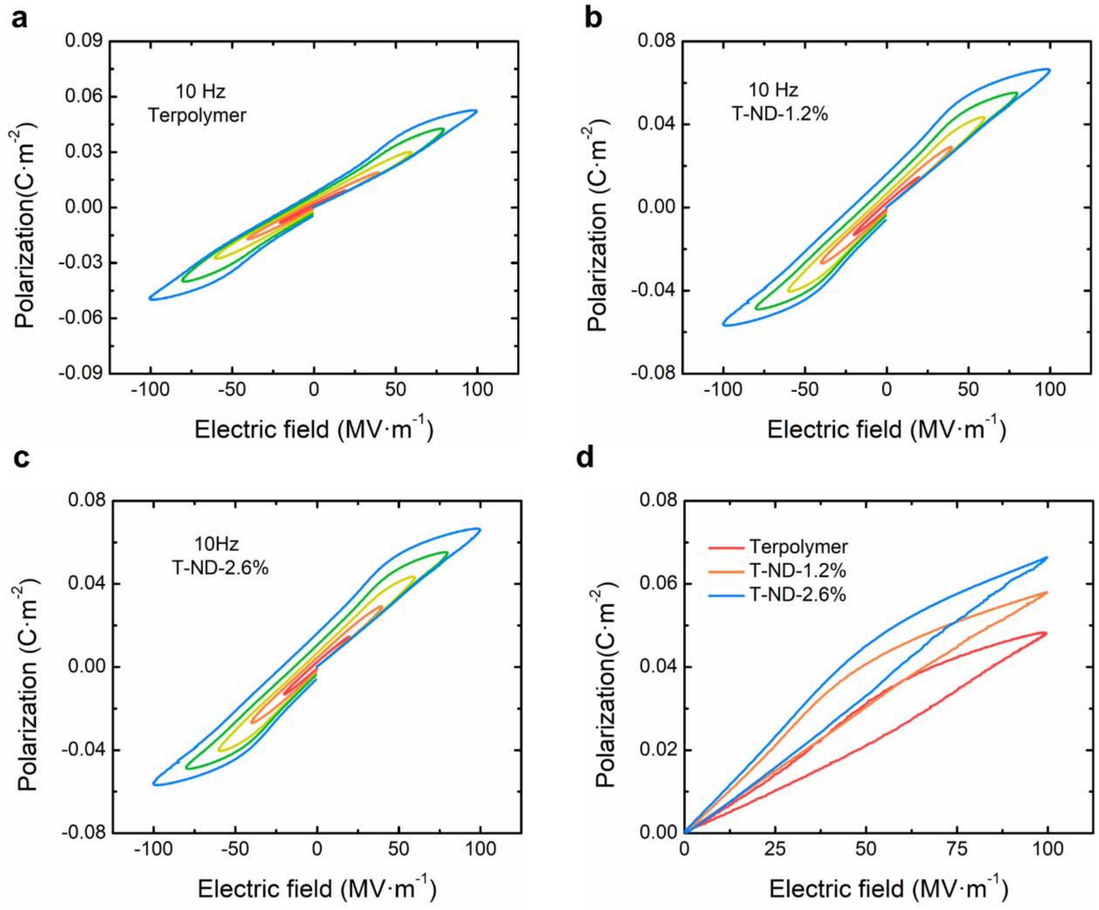

Supplementary Fig. 17. a-c P-E loops of the base terpolymer and nanocomposites. **a** terpolymer. **b** T-ND-1.2%. **c** T-ND-2.6%. **d** Comparison of the corrected P-E loops measured at 100 MV·m^{-1} and RT.

2.9. Phase-field Simulation of polarization enhancement

Here we also developed a theoretical model to verify the origin of the EC enhancement. The phase-field model was used to simulate the change in the polarization of the EC materials after the introduction of ND. The changes of the relative polarization of the nanocomposites are shown in **Supplementary Fig. 18a-b**.

This suggests that the introduction of ND particles enhances the polarization of the EC materials.

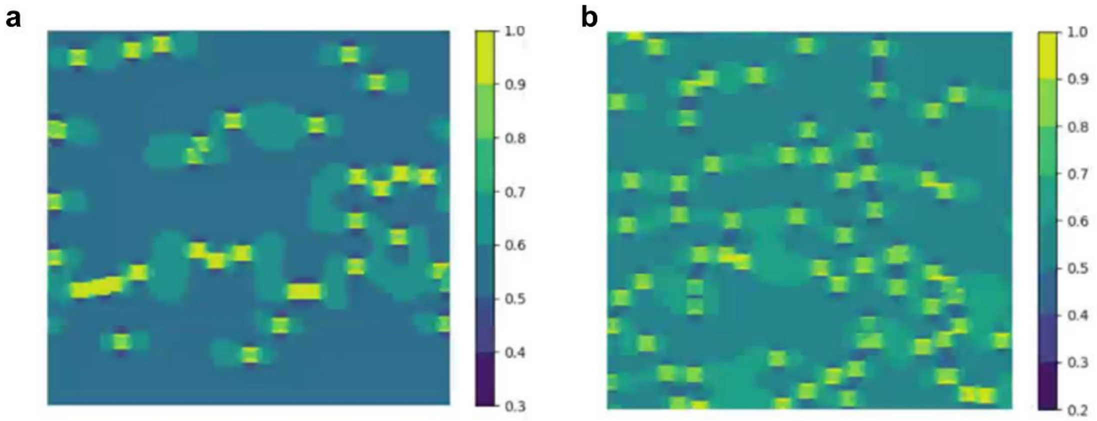

Supplementary Fig. 18. a-b Relative polarization of the ND-incorporated nanocomposites at 100 MV·m^{-1} and RT generated by the phase-field model. **a** T-ND-1.2%. **b** T-ND-2.6%.

2.10. Electrostatic Force Microscopy

To visually verify the existence of the interfacial polarization, we employed Electrostatic Force Microscopy (EFM, Park NX10) to evaluate the interfacial coupling effect of the ND-incorporated nanocomposites. It uses a scanning probe to detect changes in the electrostatic field on the sample surface, and measures the electrostatic force between the probe and the sample to obtain information about the potential distribution of the sample. Ferroelectric features were visualized by EFM with driving voltage of 1 V at a frequency of ~17 kHz.

The scanning area is a 1 μm*1 μm area containing ND-incorporated nanocomposites. The topography signal and potential signal near embedded ND were characterized, respectively, as shown in **Fig. 3a-b**.

To ensure that the ND/matrix interface in the ND-incorporated nanocomposite is well

exposed for the EFM detection, we used a two-step spin-coating preparation process[18]. First, ND (5~10 nm diameter in average) are spin-coated on an Si substrate from a dilute solution (0.005 $mg \cdot mL^{-1}$) of dimethylformamide. Subsequently the base terpolymer P(VDF-TrFE-CFE) dissolved in DMF (0.1 $mg \cdot mL^{-1}$) is spin-coated on top of the ND particles. The thickness of resultant polymeric film is dependent on the experimental parameters, and is intentionally made thinner than the height of ND particles to expose the latter.

The difference in potential signal is caused by the difference (particle-interfacial region-base terpolymer) in dielectric properties (polarization) of the materials. For EFM, the increased potential in the interfacial region detected by EFM can be caused by multiple reasons. The major two are (1) large enhancement of local dielectric constant that influences the electrostatic force[18, 19]; (2) change of surface charges[20]. Along with X-ray diffraction exhibiting no polar phase, we can exclude the formation of remnant polarization (ferroelectric polarization) in the interfacial region. Therefore, the enhancement of surface potential in EFM measurements should be ascribed to enhancement of dielectric constant or trapped space charges. Both of them can lead to the enhanced polarization as we observed in the Polarization-Electric field measurements.

2.11. Morphology of dispersed diamond nanoparticles and nanocomposite

Although the WAXD and SAXS provided macroscopic evidence for disrupting the crystalline sizes, we conducted different microscopic characterizations to provide more information on the microstructures.

ND was dispersed and then dropped onto a copper grid, followed by TEM testing. As shown in **Supplementary Fig. 19a-b**, individual ND ranges in size from 5~10 nm.

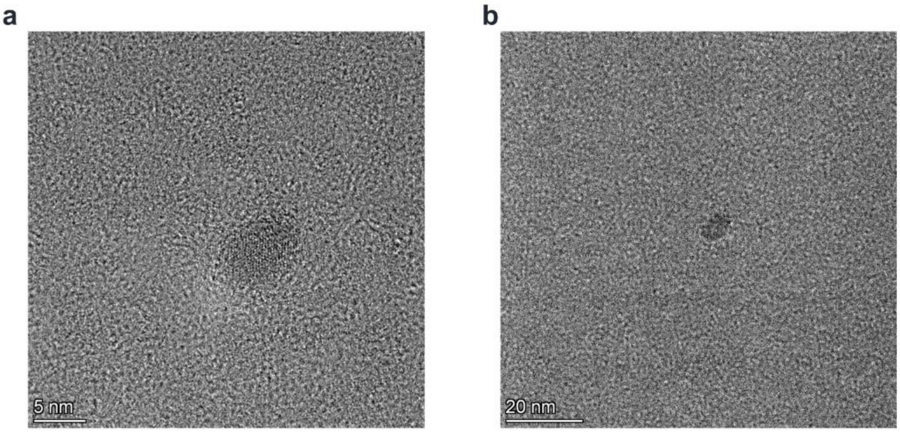

Supplementary Fig. 19. a-b TEM images of individual ND (5 nm bar and 20 nm bar).

After 8 hours of ultrasonic dispersion, the ND dispersion was dried on the substrate and ND was found to be relatively uniformly spread on the substrate, as shown in **Supplementary Fig. 20a**. More SEM images of the base terpolymer and nanocomposite films can be seen in **Supplementary Fig. 20b-c**. The base terpolymer we prepared is morphologically similar to the results in other papers[9, 15]. In addition, the morphology of the nanocomposite shows that ND was uniformly dispersed in the polymeric matrix.

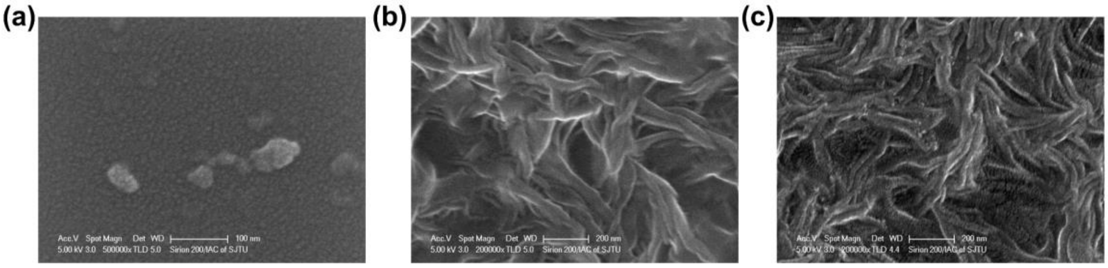

Supplementary Fig. 20. a SEM images of the dispersed ND on the silicon substrate. **b** SEM of the base terpolymer (200 nm bar). **c** SEM of T-ND-2.6% (200 nm bar).

When the NDs are dispersed into the polymer, some of the NDs will inevitably agglomerate together due to size effect. The TEM results for T-ND-2.6% are shown in

Supplementary Fig. 21. That's to say, in the nanocomposite films, there are individually dispersed NDs and also several particles agglomerated together (the size is 20~100 nm).

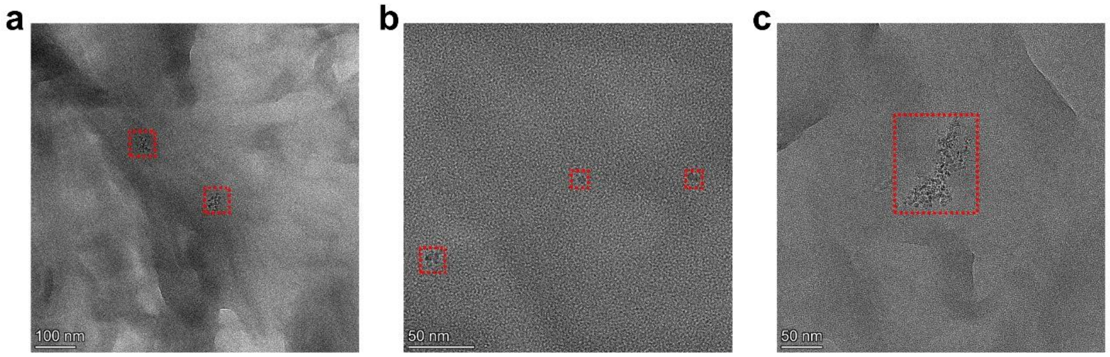

Supplementary Fig. 21. TEM images of T-ND-2.6%. **a-b** individually distributed and **c** agglomerated NDs observed inside the polymeric matrix.

2.12. The simulation of electric field distortion

We used the finite element software COMSOL 5.5 to simulate the distribution of electric field lines in the composite material under an electric field. In the simulation, the diameter of the particle is 50 nm, surrounded by the base terpolymer, and the entire area is a square with a side length of 100 nm. Here the dielectric constant of the base polymer is set to 42.

The low-k filler chosen here is ND, which has a dielectric constant near room temperature of about 6. And the high-k filler is BST, with a dielectric constant near room temperature of about 1000. A voltage of 10V was applied to both ends of the nanocomposite (*i.e.,* an electric field strength of 100 $MV{\cdot}m^{-1}$). The electric field distortion around a spherical filler with different permittivity are shown in **Supplementary Fig. 22**.

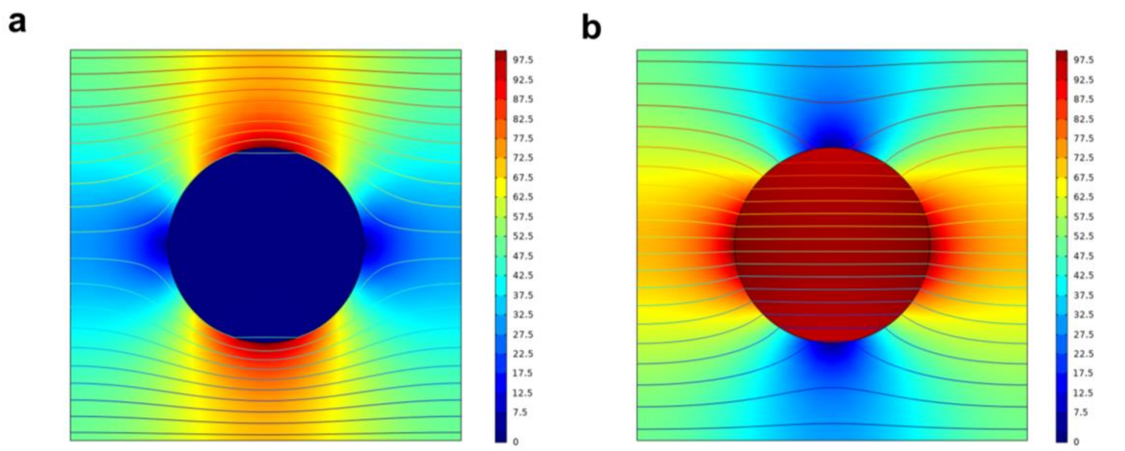

Supplementary Fig. 22. Schematic electric field distortion around a spherical filler with a permittivity higher **(a)** or lower **(b)** than the polymeric matrix. The applied electric field is 100 MV·m^{-1}.

2.13. The ECE of BNNS-incorporated nanocomposites

BNNS is known for its high thermal conductivity owing to the highly ordered 2-D atomic structure. As a result, the BNNS-incorporated P(VDF-TrFE-CFE) were expected to show smaller permittivity and reduced polarization[9]. This reduction of dielectric responses is not due to the reduction of the polar correlation in a high polar-entropy polymer[15], which is in favor of EC enhancement, but is more likely due to the BNNS-induced ordering near the polymer chain-BNNS interface under the zero-field, which could dramatically reduce the field-induced entropy change. The reduced polarization was the hint of the EC reduction that could also be predicted by the Landau phenomenological theory.

To verify the hypothesis that BNNS will deteriorate the ECE of terpolymer, we also prepared composites by blending P(VDF-TrFE-CFE) with BNNS and tested the electrocaloric cooling performance using the direct method, as shown in **Supplementary Fig. 23.**

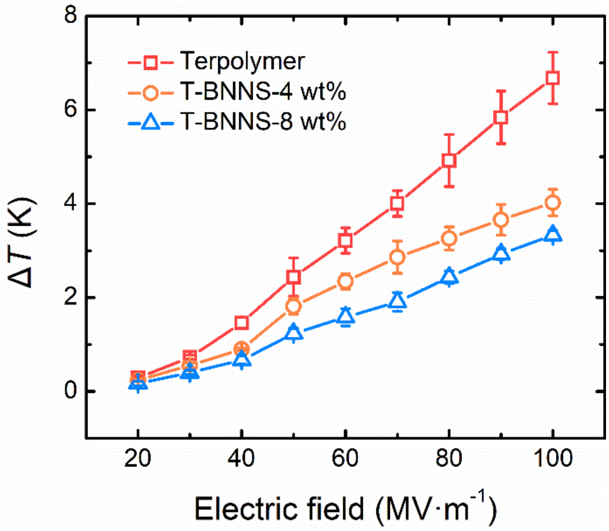

Supplementary Fig. 23. Comparison of the ECE of the base terpolymer and BNNS-incorporated nanocomposites. Sample quantities n ≥ 3, points are centered on the mean, and the bars indicate ±SD.

The ECE in those nanocomposites were markedly reduced as predicted. Therefore, we believe that blending the polymer with BNNS will deteriorate its electrocaloric cooling performance. This conclusion is also consistent with the results in the literature[9].

2.14. Landau-Devonshire phenomenological theory

ECE in an electrical insulating material can be estimated from Landau-Devonshire (L-D) phenomenological theory[21]. In a ferroelectric material, the Gibbs free energy can be written as an expansion utilizing polarization as the order parameter,

$$G = G_0 + \frac{1}{2}\alpha P^2 + \frac{1}{4}\xi P^4 + \frac{1}{6}\zeta P^6 + \cdots - EP \qquad \text{(Eq. S8)}$$

where P is the polarization, $\alpha=\beta(T-T_0)$, and β, ξ and ζ are phenomenological coefficients that are temperature independent.

In polar materials, the Gibbs free energy can be written as[21]

$$G = U - TS - X_i x_i - E_j D_j \qquad \text{(Eq. S9)}$$

where U is the internal energy, T the temperature, S the entropy, X the stress, x the strain, E the electric field and D the electric displacement. Einstein notation i runs from 1 to 6

and j from 1 to 3.

Written in the differential form, the Eq. S9 is

$$dG = -SdT - x_i dX_i - D_j dE_j \qquad \text{(Eq. S10)}$$

For most electrocaloric (EC) materials investigated, $P \approx D$. Due to $S = -\left(\partial G / \partial T\right)_{E,X}$, one obtains the isothermal entropy change of the system ΔS and the adiabatic temperature change ΔT_{EC} under an electric field E_H and E_L=0[22],

$$\Delta S = -\frac{1}{2}\beta(P^2(E_h) - P^2(E_l)) = -\frac{1}{2}\beta P^2 \qquad \text{(Eq. S11)}$$

We obtained the β coefficient by utilizing the experimental data (maximum polarization and the measured entropy changes), aiming to evaluate how efficient the overall polarization in generating ECE.

2.15. Performance at high particle content

In addition to our studies on ND low fill, we conducted performance tests with a ND volume fraction of 5.6%. We observed an increasing thermal conductivity of the ND-incorporated nanocomposites with an increasing the content of the ND, as shown in **Supplementary Fig. 24**.

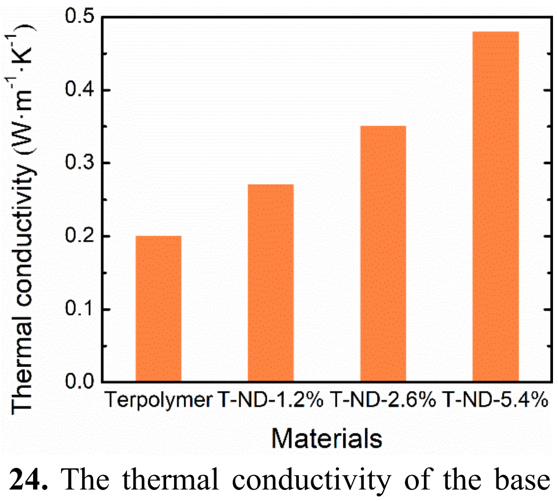

Supplementary Fig. 24. The thermal conductivity of the base terpolymer and ND-incorporated nanocomposites.

Different trend from the thermal conductivity, further increasing in NDs fillers (T-ND-5.4%) will lead to a sharp decrease in the ECE (see **Supplementary Fig. 25**) of the ND-incorporated nanocomposites due to more pronounced agglomeration (see **Supplementary Fig. 26**). The effective interfacial region induced by ND particles is the key to enhance the effective polarization and thus improve the ECE of the nanocomposites. However, this does not necessarily mean higher filler content will render better EC performances. The reasons can be summarized as:

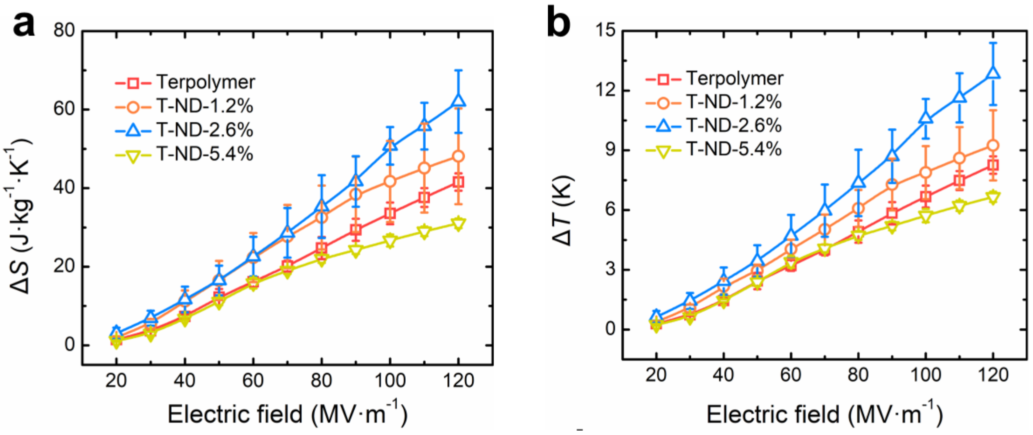

Supplementary Fig. 25. The ECE of the base terpolymer and ND-incorporated nanocomposites. **a** EC-induced ΔS. **b** EC-induced ΔT. Sample quantities n ≥ 4, points are centered on the mean, and the bars indicate ±SD.

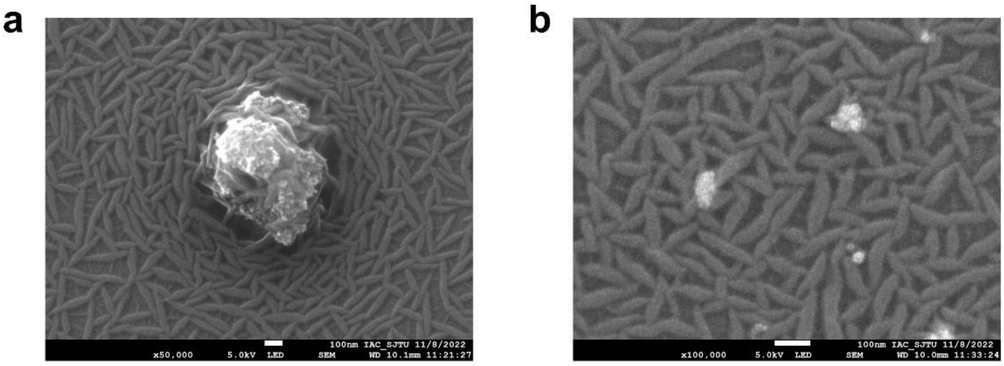

Supplementary Fig. 26. SEM of NDs. **a** agglomerated distribution in T-ND-5.4%. **b** Even distribution in T-ND-2.6%.

(1) The greatly enhanced conduction loss with high filler contents. The conduction loss of a dielectric polymer nanocomposites can be sharply enhanced with the increase

of filler content, leading to a large sacrifice in EC cooling capacity. As shown in **Supplementary Fig. 27**, compared to Terpolymer and T-ND-2.6%, T-ND-5.4% exhibits a significant Joule heating caused by the conduction, this would lead to a shift of baseline (gap between two equilibrium states). Once the electric fields are removed, the EC cooling will first compensate the residual Joule heating flux and then form a negative peak with a reduced intensity. Therefore, although adding more fillers might provide more particle surface, the highly increased conduction loss would sharply reduce the effective cooling performance of the EC nanocomposites.

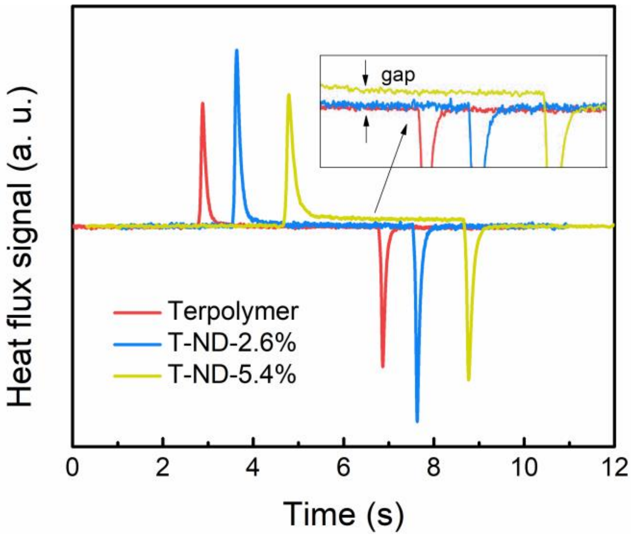

Supplementary Fig. 27. Heat flow intensity of the base terpolymer and ND-incorporated nanocomposites.

(2) The reduce of effective interfacial layer at higher filler contents. It is intuitively thought that higher filler content would give more interfacial region, thus facilitating the interfacial effect. However, recent studies have demonstrated that the dielectric constant (or polarization) were increased in a diffused layer surrounding the particles with thickness ranging from tens to hundreds of nanometers, in which the dielectric constant normally distributed[23-25]. Excessive fillers would lead to the overlap of the maximized dielectric constant region between every two particles, resulting in a decay

of interfacial effect then less enhanced polarization2. This decay is even more obvious when particles undergo severely agglomeration (vanishment of the diffused layer). In this work, we observed a similar phenomenon (see **Supplementary Fig. 28**), the effective polarization reaches the maximum in T-ND-2.6% then decays at T-ND-5.4%. The SEM images also suggest the particles in T-ND-5.4% are more likely to aggregate into large clumps (see **Supplementary Fig. 26**). This result matches well with the newly developed dielectric constant model in polymer composites[23,24]. In other words, the decrease of effective polarization at T-ND-5.4% leads to the decrease of ECE. These two interpretations also validate the strong interfacial effect in ND-incorporated nanocomposites.

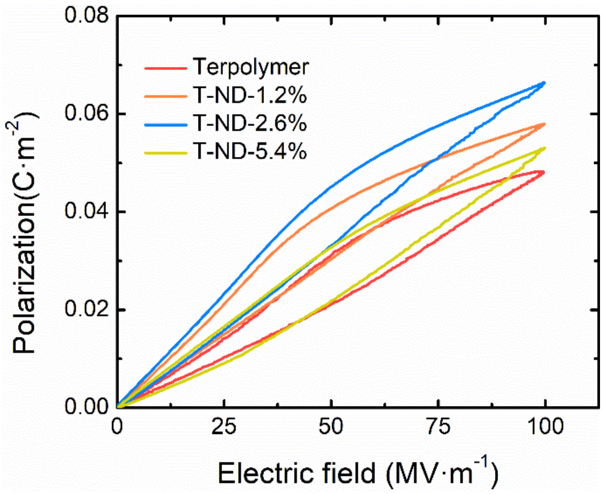

Supplementary Fig. 28. The high-field polarization of the base terpolymer and ND-incorporated nanocomposites.

3. Numerical evaluation of the rotary EC refrigeration devices

3.1. System structure and working principles

Here we introduce a rotary EC device, featuring rotary EC components, a motor, heat exchangers, pumps, and other pertinent elements. The rotary EC module is a cylindrical holder meticulously designed to accommodate layers of EC materials. Propelled by a motor, as delineated in **Fig 5a**, the cylindrical holder for the EC assembly boasts precise dimensions—a 4 mm inner diameter, an 11.1 mm outer diameter, and a 50 mm height. Uniformly divided, it accommodates four EC working parts for even distribution. Each individual EC part within the holder holds 2.135 cm^3 of EC materials, resulting in a total volume of 8.54 cm^3 across all four parts. The singular EC working part comprises 36 polymer sheets (nanocomposites) spaced at 100 μm intervals, maintaining the same spacing between any two adjacent EC films within the part.

As a segment of the EC materials exits the electric field zone, demarcated by the shaded rectangle area in **Fig. 5a**, a corresponding section of EC materials enters this region. This arrangement is designed to ensure that a portion of the EC materials remains in the cooling state, facilitating continuous cooling. Upon entering the electric field zone, the temperature of the EC materials rises, subsequently elevating the temperature of the heat transfer fluid. Throughout the rotation process, a thermal equilibrium is established between the EC materials and the heat transfer fluid. The hot heat transfer fluid is then extracted by a pump on the hot side, transferring heat to the external environment through the hot-side heat exchanger. A phase of static heat exchange occurs between the fluid entering the EC part and the EC materials before the

EC part is about to exit the electric field zone. The operational strategy of the rotary EC part post-departure from the electric field zone mirrors that employed while within the zone. Cold fluid is directed to the cold-side heat exchanger, delivering the necessary cooling effect where required. By perpetuating these operational processes, the system can consistently provide a continuous cooling capacity[28].

To streamline computational resources, a singular EC part is strategically chosen and discretized into a mesh. The insulation of the EC part from the external environment is deliberately designed, thus negating the need to consider heat transfer between the unit and its surroundings throughout the computational process. The fluid flow within the EC part is presumed to be incompressible, and its dynamics are accurately modeled using the Navier-Stokes equations. Furthermore, a heat transfer fluid circulates within the system, facilitating the exchange of heat with the EC materials present in the designated EC working part. Below is reported the structure of the mathematical model that describes the flow and heat transfer process[2].

$$\frac{\partial u}{\partial x}+\frac{\partial v}{\partial y}+\frac{\partial w}{\partial z}=0 \qquad \text{(Eq. S12)}$$

$$\frac{\partial u}{\partial t}+u\frac{\partial u}{\partial x}+v\frac{\partial u}{\partial y}+w\frac{\partial u}{\partial z}=-\frac{1}{\rho_{\mathrm{f}}}\frac{\partial p}{\partial x}+\upsilon\left(\frac{\partial^2 u}{\partial x^2}+\frac{\partial^2 u}{\partial y^2}+\frac{\partial^2 u}{\partial z^2}\right) \qquad \text{(Eq. S13)}$$

$$\frac{\partial v}{\partial t}+u\frac{\partial v}{\partial x}+v\frac{\partial v}{\partial y}+w\frac{\partial v}{\partial z}=-\frac{1}{\rho_{\mathrm{f}}}\frac{\partial p}{\partial y}+\upsilon\left(\frac{\partial^2 v}{\partial x^2}+\frac{\partial^2 v}{\partial y^2}+\frac{\partial^2 v}{\partial z^2}\right) \qquad \text{(Eq. S14)}$$

$$\frac{\partial w}{\partial t}+u\frac{\partial w}{\partial x}+v\frac{\partial w}{\partial y}+w\frac{\partial w}{\partial z}=-\frac{1}{\rho_{\mathrm{f}}}\frac{\partial p}{\partial z}+\upsilon\left(\frac{\partial^2 w}{\partial x^2}+\frac{\partial^2 w}{\partial y^2}+\frac{\partial^2 w}{\partial z^2}\right) \qquad \text{(Eq. S15)}$$

Their energy relationship satisfies the following equation:

$$\frac{\partial T_{\mathrm{f}}}{\partial t}+u\frac{\partial T_{\mathrm{f}}}{\partial x}+v\frac{\partial T_{\mathrm{f}}}{\partial y}+w\frac{\partial T_{\mathrm{f}}}{\partial z}=\frac{k_{\mathrm{f}}}{\rho_{\mathrm{f}}c_{\mathrm{f}}}\left(\frac{\partial^2 T_{\mathrm{f}}}{\partial x^2}+\frac{\partial^2 T_{\mathrm{f}}}{\partial y^2}+\frac{\partial^2 T_{\mathrm{f}}}{\partial z^2}\right) \qquad \text{(Eq. S16)}$$

$$\frac{\partial T_s}{\partial t} = \frac{k_s}{\rho_s c_s}\left(\frac{\partial^2 T_s}{\partial x^2} + \frac{\partial^2 T_s}{\partial y^2} + \frac{\partial^2 T_s}{\partial z^2}\right) + \frac{Q}{\rho_s c_s} \qquad \text{(Eq. S17)}$$

The source term Q in Eq. (S6) converts the caloric effect into a power density, which is a function of the field and temperature[27]. The source term Q can be calculated as:

$$Q = Q(E, T_s) = \frac{\rho_s c_s \Delta T(E, T_s)}{\tau} \qquad \text{(Eq. S18)}$$

The EC effect of the base terpolymer and nanocomposites exhibits a small deviation when the ambient temperature is from 280 K to 320 K, as is shown in **Fig.4a-b**. For reasons of simplicity of calculation, the entropy changes and adiabatic temperature changes are considered as temperature independent within the temperature range.

3.2. Initial and boundary conditions

The application of a moving-mesh method efficiently addresses the dynamically evolving boundary conditions within the system, operating at a frequency of 4 Hz. Initially, a constant internal heat source term, denoted as Q, is imposed within the solid EC materials. This internal heat source persists for a duration of 0.025 s, aligning with the period during which the EC part is exposed to the electric field.

Subsequent to half a cyclic period (0.125 s), a negative internal heat source term is introduced into the EC materials, coinciding with the moment the EC part exits the electric field. Upon the EC part entering the electric field zone and during its subsequent rotation, a phase of static heat exchange occurs. In this phase, the heat transfer fluid and EC materials within the EC part remain relatively stationary, rotating counterclockwise. Following this, the heat transfer fluid enters the EC part at a defined speed (0.5 $m \cdot s^{-1}$) and temperature (T_C) from one side of the inlet, while the opposite side serves as the

pressure outlet (101325 Pa). This process involves the heating of the fluid by the EC materials, followed by the discharge of the heated fluid. **Supplementary Fig. 29** provides a schematic representation of the detailed mesh and boundary conditions.

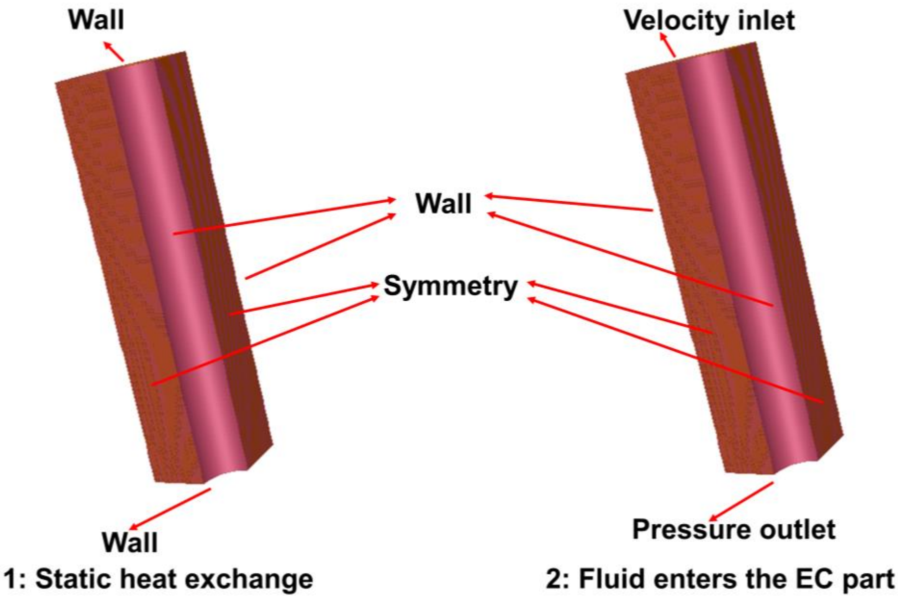

Supplementary Fig. 29. Boundary conditions of two working stages and local grid diagram.

Then the fluid is cooled by the EC materials leaving the electric field, while the cold fluid is expelled from the EC part in a similar process (At this period the inlet temperature is T_H). The thermal-insulated boundary conditions are currently used to solve the contact problem of the individual component with others.

In this study, mesh-independence verification and selection of a suitable mesh can help speed up the computational process. Models with grid numbers of 282640, 1573000, 3714000 and 6842000 were constructed. As shown in **Supplementary Fig. 30**, the grid-independent validation results show that when the number of grids exceeds 3714000, the average temperature of the cold fluid outlet (T_{cold}) hardly changes anymore (less than 0.1 K). Therefore, the model with the grid number of 3714000 was

chosen for the calculation.

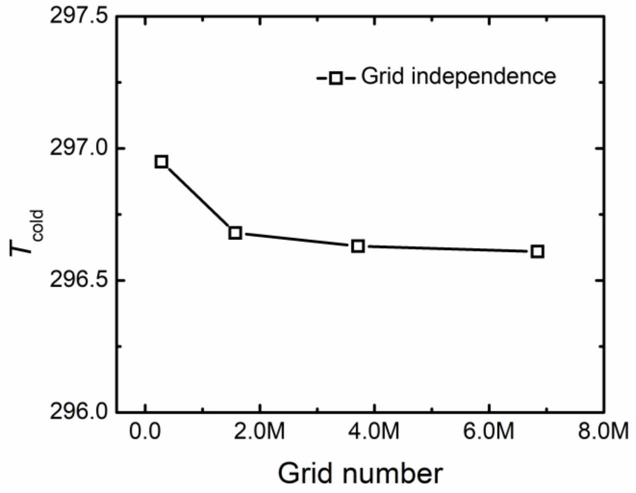

Supplementary Fig. 30. Grid-independent verification.

3.3. Numerical evaluation of the rotary EC System

The evaluation of material thermal penetration depth is an important factor for judging whether the system has sufficient heat exchange[26]. The thermal penetration depth, δ, which is given by the following Eq. S8. Improving the thermal conductivity of EC materials can help increase the thermal penetration depth (δ), or furthermore, guarantee the refrigeration system can better operate at higher frequencies. Under the designed working frequency, the corresponding δ of various materials are shown in **Supplementary Table 2**. Among the terpolymer and nanocomposites, it's obvious that T-ND-2.6% has the best heat transfer performance, followed by T-ND-1.2%. For the current EC device, T-ND-2.6% as core components can meet the need for adequate heat exchange (δ >100 μm).

$$\delta = \sqrt{\frac{\lambda}{\pi f \rho c}} \qquad \text{(Eq. S19)}$$

where f is the operating frequency of the EC device and λ, q, and c are the thermal

conductivity, density, and specific heat of the key materials used in the EC device.

Supplementary Table 2. Comparison of δ corresponding to different materials.

Materials	Terpolymer	T-ND-1.2%	T-ND-2.6%	Deionized water
δ (μm)	76.8	89.3	101.6	106.9

The temperature distribution of the heat transfer fluid and EC materials in the cycle of refrigeration devices using different EC materials over a 10 K temperature span is shown in the **Supplementary Fig. 31**. The graph shows that the cold fluid outlet temperature is lowest for the device using T-ND-2.6% and highest for the device using base terpolymer as core components. This can also help to differentiate the cooling performance of different EC devices under the same operating conditions.

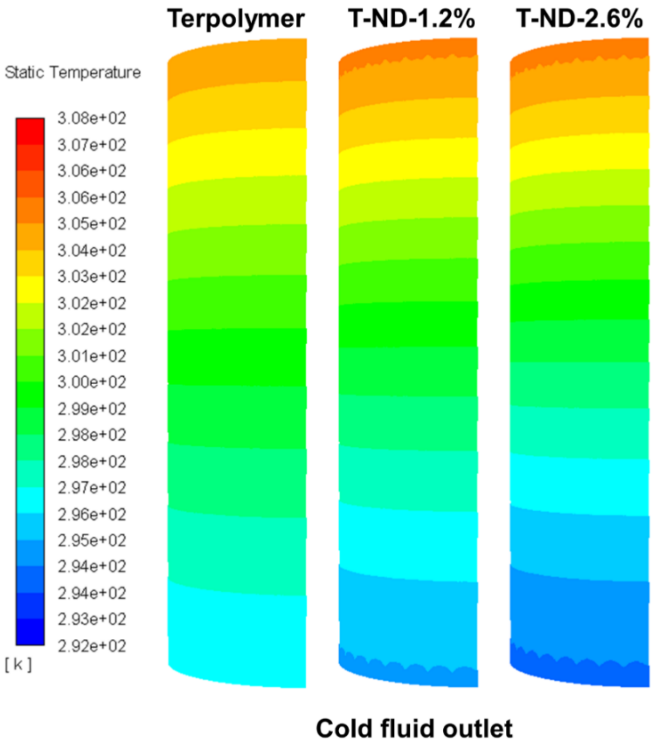

Supplementary Fig. 31. Temperature distribution of the working part based on different kinds of EC materials at the same time (2.45 s).

4. EC device as a standard platform

4.1. Electrocaloric cooling with electrostatic actuation

We developed a film-like EC oscillating refrigerator, adopting a configuration and operational strategy akin to the previously reported EC device[13]. This standardized platform facilitates the evaluation of material properties and their interplay with device performance. **Supplementary Fig. 32a-b** provide detailed side views of the device. The EC film is situated between two surface-insulated stainless-steel sheets, subject to alternating electrostatic forces driven by changes in the applied electric field. Behind these sheets, two ceramic plates, maintained at constant temperatures, serve as the hot and cold ends. Two initially calibrated heat flux sensors are strategically placed between these plates to precisely capture heat flux signals.

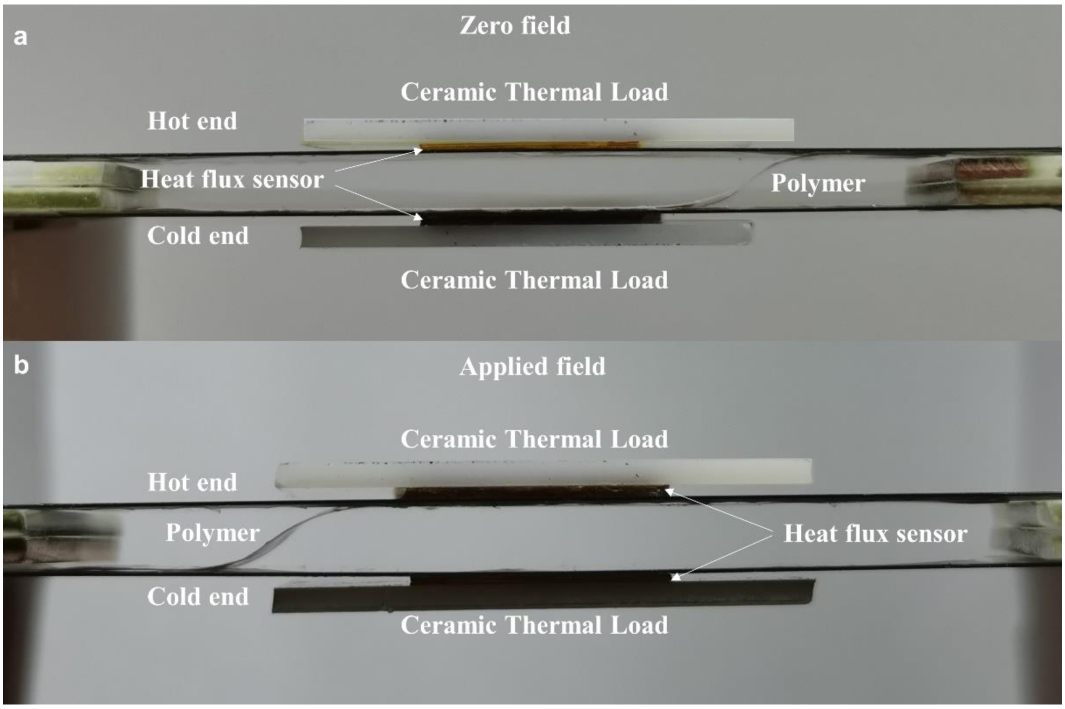

Supplementary Fig. 32. The side views of our EC device under **(a)** Zero field and **(b)** Applied field.

4.2. Comparison of device performance

Supplementary Fig. 33 delineates the intricate procedures involved in calibrating

and measuring the heat flux signals. To calibrate the sensors, a constant resistance (*R*) heating plate was employed, as illustrated in **Supplementary Fig. 33**. The resistance was positioned on the ceramic plate, with the sensor situated between them, and thermal insulation foam was applied over the resistance. Initially, the sensor recorded a stable signal denoted as U_0. As a voltage (U) was applied to the resistance, Joule heat ($P_{Joule}=U^2/R$) was transferred from the resistance to the ceramic plate through the sensor, causing a changed signal U_1. We got the change value in voltage ($U_{change}=U_1-U_0$) of the sensor proportional to the Joule heat as the signal was stable, and the proportional factor γ between heating power density and sensor signal could be calculated as $\gamma=PJ_{oule}/(S*U_{change})$ in which S is the area of the resistance.

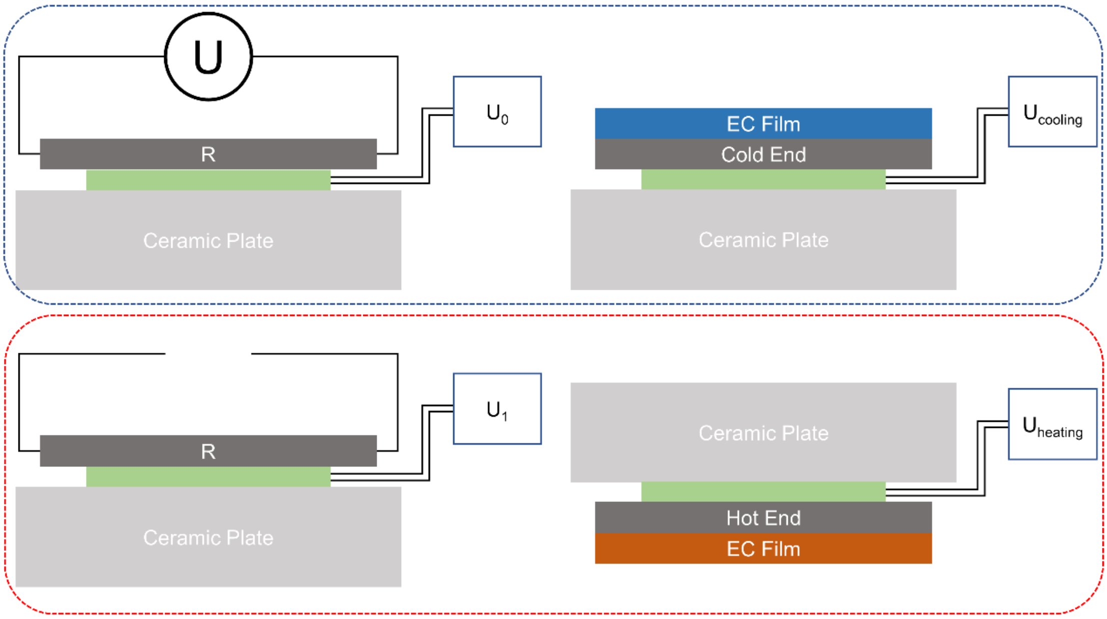

Supplementary Fig. 33. The calibration process for heat flux sensors in the EC device.

In the device, as the EC film alternatively attached to cold and hot ends as shown in **Supplementary Fig. 32**, signals appeared as $U_{cooling}$, and the original signals are provided in **Supplementary Fig. 34**. The corresponding heating (HPD) and cooling

(CPD) power densities could be calculated as HPD=γ*$U_{heating}$/(t_h*ρ) and CPD=γ*Ucooling/(t_h*ρ) in which t_h is the thickness of the EC film (30 μm) and ρ is the density.

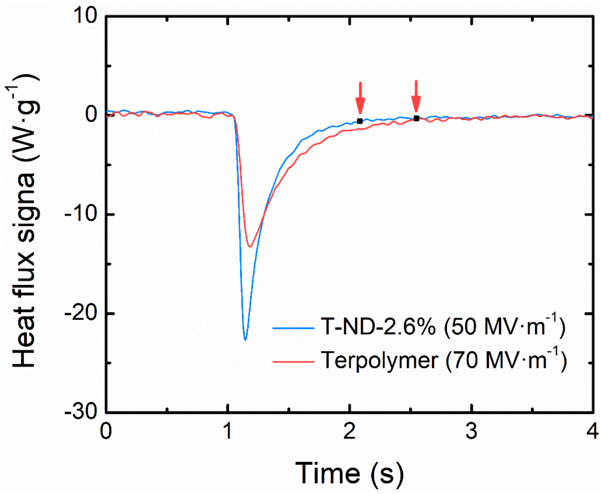

Supplementary Fig. 34. The heat flux signal measurement in the EC device.

The experimental results of this platform are intended to illustrate the superior cooling capacity (the same CPD can be obtained at lower electric fields) and the better heat transfer performance (shorter heat dissipation time) of the ND-incorporated nanocomposites compared to the base terpolymer.

References

1. Hu H, Zhang F, Luo S, Yue J, Wang CH. Electrocaloric effect in relaxor ferroelectric polymer nanocomposites for solid-state cooling. *Journal of Materials Chemistry A* 8, (2020).

2. Li Q, Shi J, Han D, Du F, Chen J, Qian X. Concept design and numerical evaluation of a highly efficient rotary electrocaloric refrigeration device. *Applied Thermal Engineering* 190, (2021).

3. Qian X. Pumping into a cool future: electrocaloric materials for zero-carbon refrigeration. *Frontiers in Energy* 16, 19-22 (2022).

4. Defay E*, et al.* Enhanced electrocaloric efficiency via energy recovery. *Nat Commun* 9, 1827 (2018).

5. Shi J*, et al.* Electrocaloric Cooling Materials and Devices for Zero-Global-Warming-Potential, High-Efficiency Refrigeration. *Joule* 3, 1200-1225 (2019).

6. Qian X-S*, et al.* Giant Electrocaloric Response Over A Broad Temperature Range in Modified BaTiO3Ceramics. *Advanced Functional Materials* 24, 1300-1305 (2014).

7. Plaznik U*, et al.* Bulk relaxor ferroelectric ceramics as a working body for an electrocaloric cooling device. *Applied Physics Letters* 106, (2015).

8. Lu Y-C*, et al.* Enhanced electrocaloric effect for refrigeration in lead-free polymer composite films with an optimal filler loading. *Applied Physics Letters* 114, (2019).

9. Guangzu*, et al.* Ferroelectric polymer nanocomposites for room-temperature electrocaloric refrigeration. *Advanced materials (Deerfield Beach, Fla)*, (2015).

10. Qian J*, et al.* Interfacial Coupling Boosts Giant Electrocaloric Effects in Relaxor Polymer Nanocomposites: In Situ Characterization and Phase-Field Simulation. *Adv Mater* 31, e1801949 (2019).

11. Chen Y*, et al.* An All-Scale Hierarchical Architecture Induces Colossal Room-Temperature Electrocaloric Effect at Ultralow Electric Field in Polymer Nanocomposites. *Adv Mater* 32, e1907927 (2020).

12. Neese B, Chu B, Lu S-G, Wang Y, Furman E, Zhang Q. Large electrocaloric effect in ferroelectric polymers near room temperature. *Science* 321, 821-823 (2008).

13. Ma R*, et al.* Highly efficient electrocaloric cooling with electrostatic actuation. *Science*

357, 1130-1134 (2017).

14. Bai P*, et al.* An Active Pixel-matrix Electrocaloric Device for Targeted and Differential Thermal Management. *Advanced Materials*, 2209181 (2023).

15. Qian X*, et al.* High-entropy polymer produces a giant electrocaloric effect at low fields. *Nature* 600, (2021).

16. Tencé-Girault S, Lebreton S, Bunau O, Dang P, Bargain F. Simultaneous SAXS-WAXS experiments on semi-crystalline polymers: Example of PA11 and its brill transition. *Crystals* 9, 271 (2019).

17. Chu B. *PVDF-based copolymers, terpolymers and their multi-component material systems for capacitor applications*. The Pennsylvania State University (2008).

18. Simin*, et al.* Direct Detection of Local Electric Polarization in the Interfacial Region in Ferroelectric Polymer Nanocomposites. *Advanced Materials* 31, 1807722-1807722 (2018).

19. Peng S, Zeng Q, Yang X, Hu J, Qiu X, He J. Local dielectric property detection of the interface between nanoparticle and polymer in nanocomposite dielectrics. *Scientific reports* 6, 38978 (2016).

20. Schoenherr P, et al. Observation of uncompensated bound charges at improper ferroelectric domain walls. *Nano letters* 19, 1659-1664 (2019).

21. Lines ME, Glass AM. *Principles and applications of ferroelectrics and related materials*. Oxford university press (2001).

22. Lu S-G, Zhang Q. Large electrocaloric effect in relaxor ferroelectrics. *Journal of Advanced Dielectrics* 2, 1230011 (2012).

23. Li L, et al. Significant improvements in dielectric constant and energy density of ferroelectric polymer nanocomposites enabled by ultralow contents of nanofillers. *Advanced Materials* 33, 2102392 (2021).

24. Thakur Y, et al. Enhancement of the dielectric response in polymer nanocomposites with low dielectric constant fillers. *Nanoscale* 9, 10992-10997 (2017).

25. Zhang B, Chen X, Lu W, Zhang Q, Bernholc J. Morphology-induced dielectric enhancement in polymer nanocomposites. *Nanoscale* 13, 10933-10942 (2021).

26. Shi J*, et al.* Numerical evaluation of a kilowatt-level rotary electrocaloric refrigeration system. *International Journal of Refrigeration*, (2020).

27. Aprea C, Greco A, Maiorino A, Masselli C. The employment of caloric-effect materials for solid-state heat pumping. *International Journal of Refrigeration* 109, 1-11 (2020).

28. Guo D*, et al.* Design and modeling of a fluid-based micro-scale electrocaloric refrigeration system. *International Journal of Heat and Mass Transfer* 72, 559-564 (2014).
