# Supplementary figures and images for: Low-k nano-dielectrics facilitate electric-field induced phase transition in high-k ferroelectric polymers for sustainable electrocaloric refrigeration
Source: Nat Commun. 2024 Jan 24;15:702. doi: 10.1038/s41467-024-44926-8 (PMC10808131; doi:10.1038/s41467-024-44926-8)

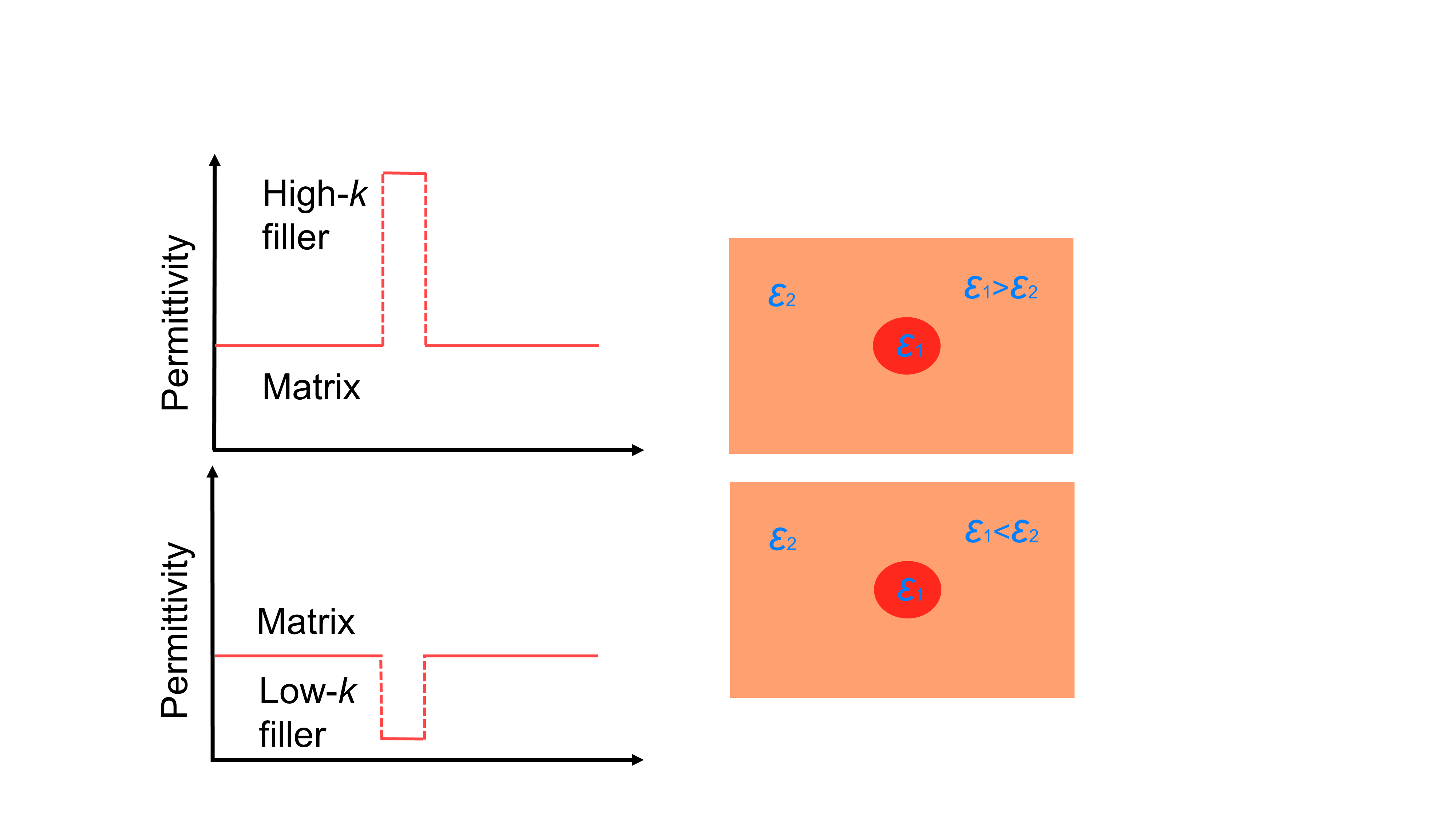

Supplement: Supplementary file 4 — Source Data [file 41467_2024_44926_MOESM4_ESM.zip › Data/Fig.1a.tif]

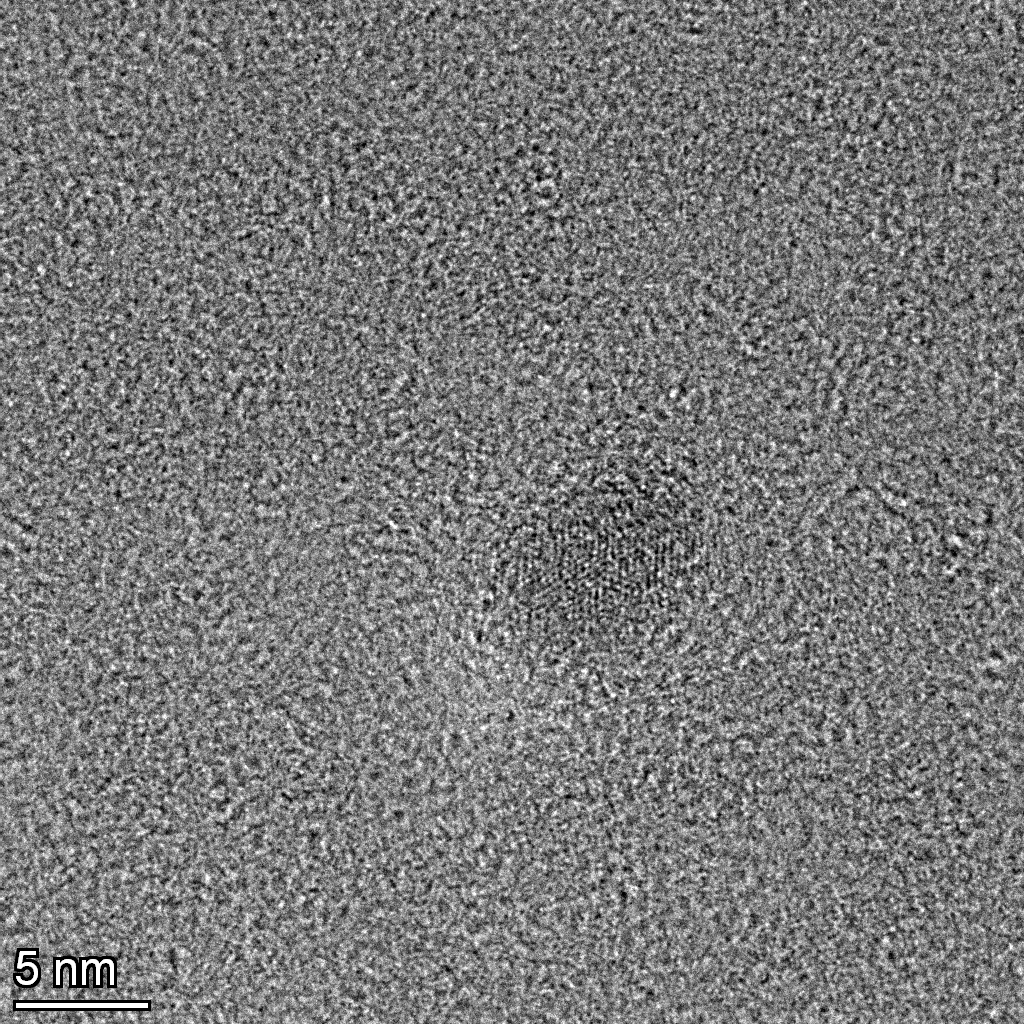

Supplement: Supplementary file 4 — Source Data [file 41467_2024_44926_MOESM4_ESM.zip › Data/Fig.1b.tif]

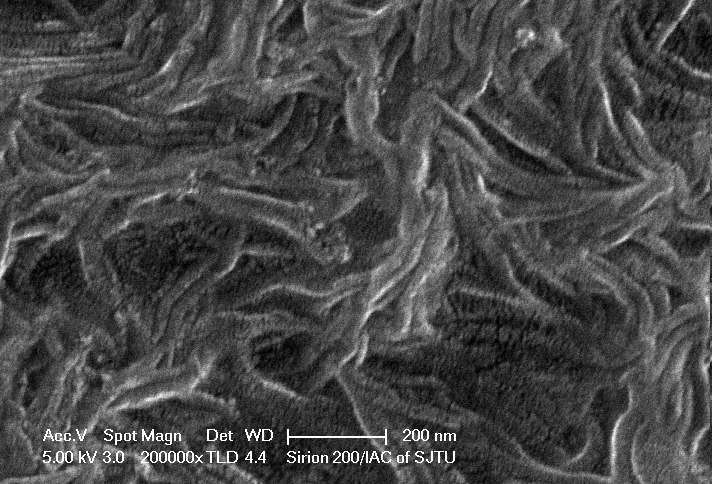

Supplement: Supplementary file 4 — Source Data [file 41467_2024_44926_MOESM4_ESM.zip › Data/Fig.1c.TIF]

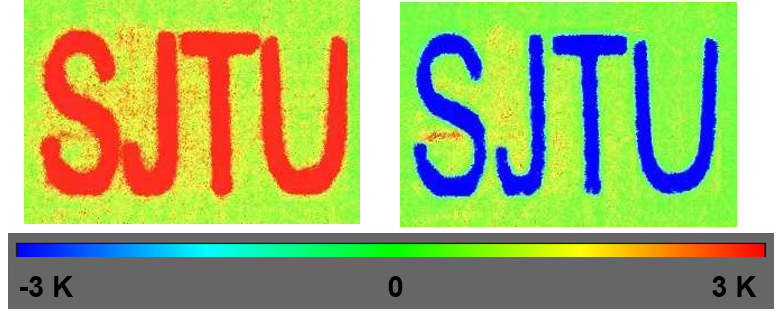

Supplement: Supplementary file 4 — Source Data [file 41467_2024_44926_MOESM4_ESM.zip › Data/Fig.1h.TIF]

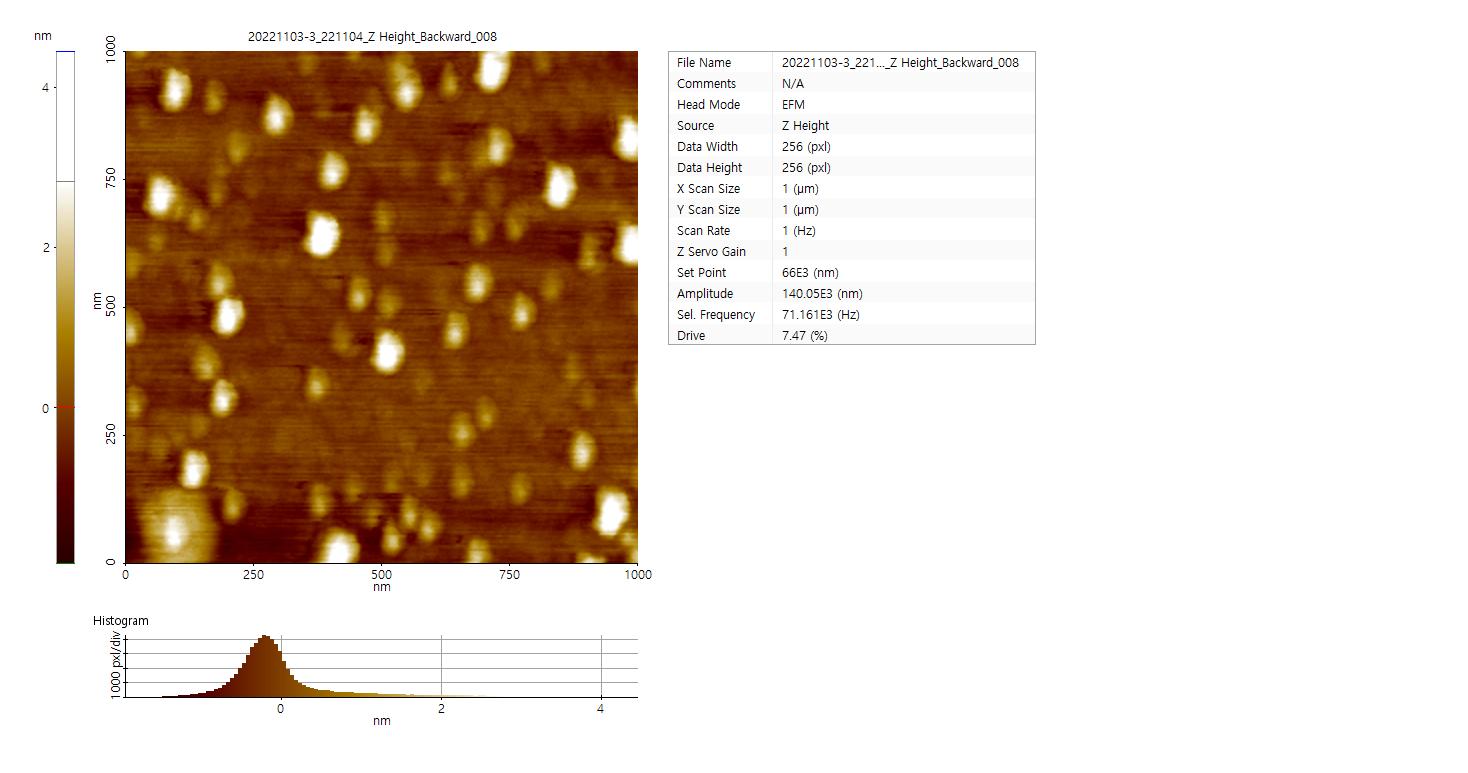

Supplement: Supplementary file 4 — Source Data [file 41467_2024_44926_MOESM4_ESM.zip › Data/Fig.3a.jpg]

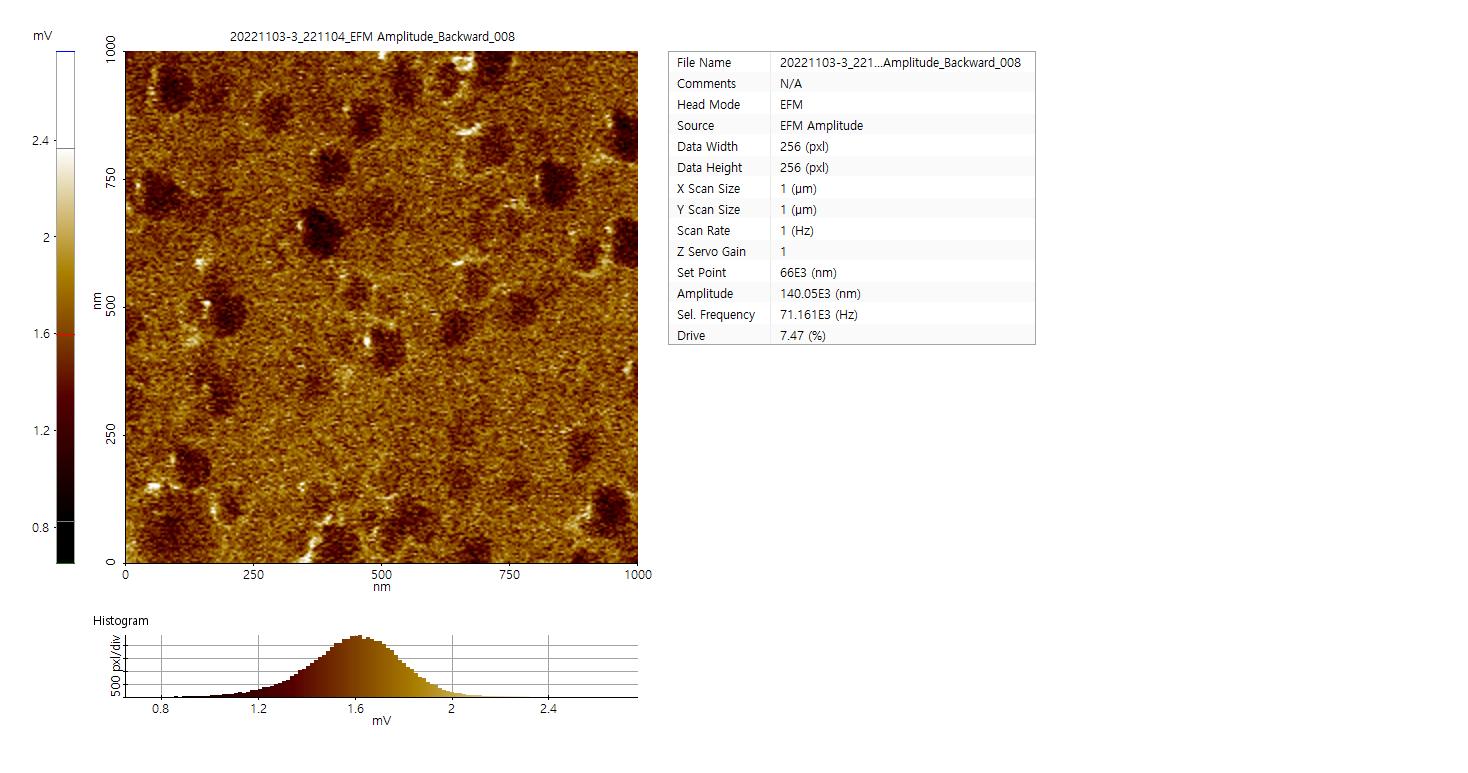

Supplement: Supplementary file 4 — Source Data [file 41467_2024_44926_MOESM4_ESM.zip › Data/Fig.3b.jpg]

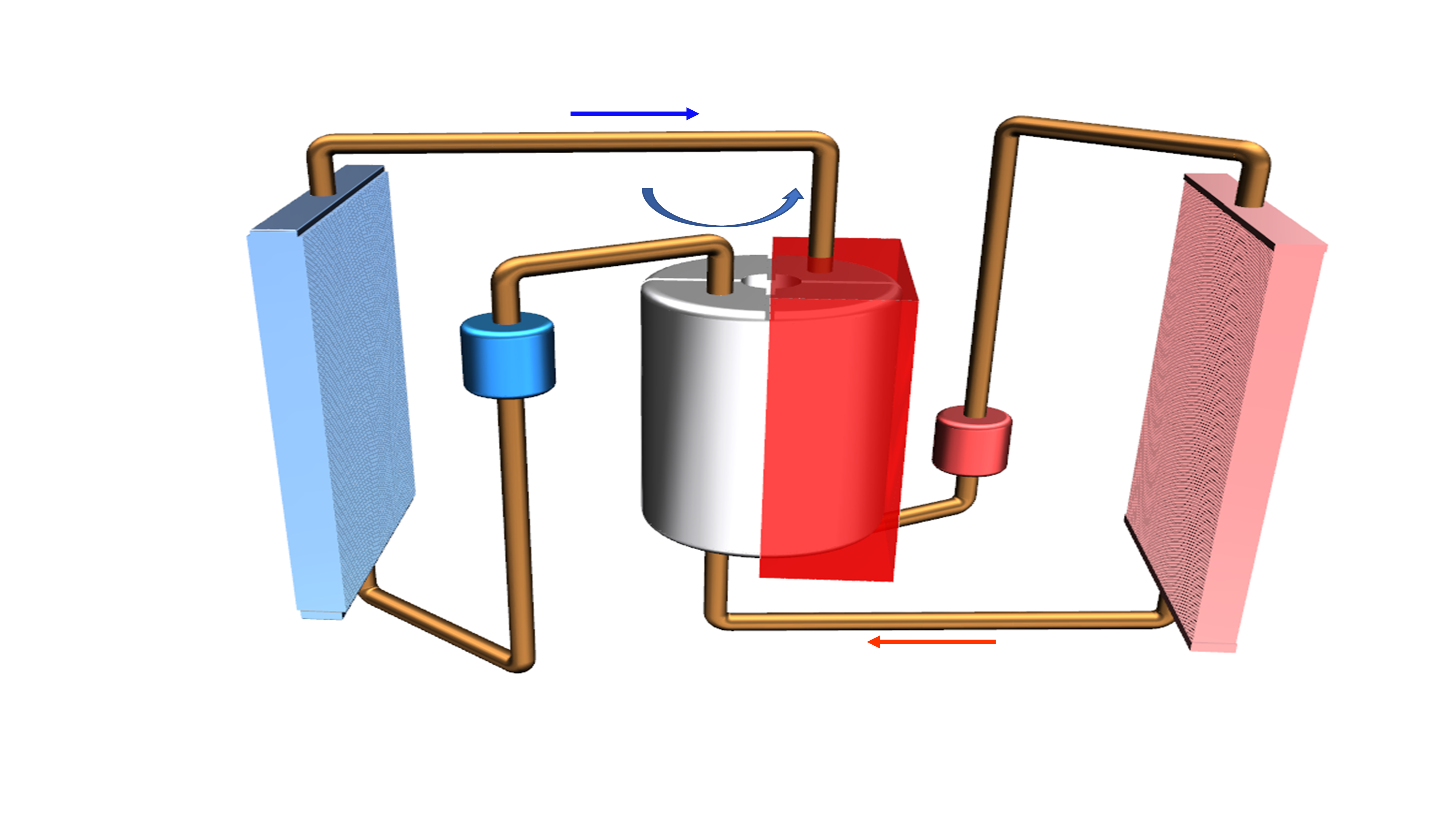

Supplement: Supplementary file 4 — Source Data [file 41467_2024_44926_MOESM4_ESM.zip › Data/Fig.5a.tif]
